# Tourism and the Conservation of Critically Endangered Frogs

**DOI:** 10.1371/journal.pone.0043757

**Published:** 2012-09-12

**Authors:** Clare Morrison, Clay Simpkins, J. Guy Castley, Ralf C. Buckley

**Affiliations:** 1 International Centre for Ecotourism Research, Griffith School of Environment, Griffith University, Queensland, Australia; 2 Environment Futures Centre, Griffith School of Environment, Griffith University, Queensland, Australia; Australian Wildlife Conservancy, Australia

## Abstract

Protected areas are critical for the conservation of many threatened species. Despite this, many protected areas are acutely underfunded, which reduces their effectiveness significantly. Tourism is one mechanism to promote and fund conservation in protected areas, but there are few studies analyzing its tangible conservation outcomes for threatened species. This study uses the 415 IUCN critically endangered frog species to evaluate the contribution of protected area tourism revenue to conservation. Contributions were calculated for each species as the proportion of geographic range inside protected areas multiplied by the proportion of protected area revenues derived from tourism. Geographic ranges were determined from IUCN Extent of Occurrence maps. Almost 60% (239) of critically endangered frog species occur in protected areas. Higher proportions of total range are protected in Nearctic, Australasian and Afrotopical regions. Tourism contributions to protected area budgets ranged from 5–100%. These financial contributions are highest for developing countries in the Afrotropical, Indomalayan and Neotropical regions. Data for both geographic range and budget are available for 201 critically endangered frog species with proportional contributions from tourism to species protection ranging from 0.8–99%. Tourism's financial contributions to critically endangered frog species protection are highest in the Afrotropical region. This study uses a coarse measure but at the global scale it demonstrates that tourism has significant potential to contribute to global frog conservation efforts.

## Introduction

The in-situ conservation of viable populations in natural ecosystems is widely recognized as a fundamental requirement for the maintenance of biodiversity [1]. Protected areas are a key component of many in-situ conservation strategies, particularly for threatened species [2], [3], [4], [5]. Currently, approximately 12% of the Earth's land surface is under some form of protection, with more than 120,000 protected areas recognized [6], [7]. Protection of biodiversity by existing protected area networks remains inadequate however because they are either too small [4], [8], [9], poorly located [4], [8], poorly protected [3], [10], or provide unsuitable habitats [4], [8], [11].

One widespread and recurrent barrier to the effective operation of protected area networks, especially in developing regions, is lack of funding [12], [13], [14], [15]. Previous studies examining the cost of effectively managing existing protected areas in developing regions identified a funding shortfall of US$1–1.7 billion [16], [17]. Furthermore, an additional US$4 billion would be needed per year over 10 years to establish and manage an expanded protected area system [13], [14], [16], [17], [18]. Regardless of these projections protected area systems, particularly in highly biodiverse developing nations, remain deficient in government funding [13], [16]. Consequently, they are increasingly having to become financially self sustaining [19], [20]. Conservation agencies at local and national levels are therefore exploring alternative funding mechanisms. Payments for ecosystem services (PES), carbon capture projects, fiscal instruments (e.g. environmental taxes), and nature-based tourism all have potential to generate revenue for protected areas [13], [21], [22], [23].

The tourism industry has become one the fastest growing global industries and contributes approximately 5% to global GDP and more than 10% to GDP in developing regions [24]. The nature-based tourism segment is a key niche market experiencing significant growth [25], and is dependent on natural settings and environments including protected areas. Visitor entrance fees, donations and commercial tourism concessions can generate revenue for protected areas at the local scale [20], [26]. Similar national level mechanisms include government visitor taxes (e.g. environment taxes) and the transfer of site-generated revenue to a central agency for total or partial redistribution amongst all protected areas. As with revenue from all sources, this funding can be used for a range of conservation management activities including habitat restoration, invasive species removal, reintroduction of threatened species and anti-poaching or logging patrols [23], .

Most previous research on the contribution of tourism to conservation has focused on socio-economic aspects [32], [33], [34], [35]. While many suggest that tourism can deliver conservation benefits, this view is not unanimous [36], and there is little direct evidence of tangible conservation outcomes. Theoretically, however, if high proportions of protected area budgets are derived from tourism, tourism makes a significant financial contribution to the management of these protected areas regardless of the activities funded i.e. salaries, infrastructure maintenance, habitat restoration, etc. The extent to which tourism revenue supports species conservation therefore requires empirical evaluation. Here we conduct such an evaluation using critically endangered (CR) frogs as our indicator taxonomic group and quantified the financial contribution of tourism to their conservation in protected areas. Frogs were selected since at the global scale, the Amphibia are more severely threatened than any other order, with 30% (1669/5584 species) listed as threatened by IUCN. This compares with 22% for mammals and 13% for birds [37]. Range data for individual threatened frog species are less complete than for mammals and birds so we focus here on only the 415 CR species which have the best data available. The results of this work allow us to quantify the contribution of tourism to the protection of threatened species. At a time when management agencies have to become increasingly self-reliant in the face of critical government funding shortages our study highlights the significance of tourism, specifically tourism generated revenue, to protected areas and the threatened species supported by these areas.

## Methods

We calculated the contribution of tourism to the conservation of each CR frog species in three successive steps. First we identified the global range for each species and calculated the proportion of its range within protected areas (PAs) using GIS analysis. Secondly, we obtained financial information on the proportion of PA operating budgets derived from tourism in each country. Thirdly, we calculated the financial contribution of tourism to the protection and conservation of each frog species as the product of the first two parameters.

### Critically endangered frogs and protected areas

We identified critically endangered frogs using the IUCN Red List of Threatened Species [37] for assessments from 2003–2011. We extracted all species records and compiled a database of country/countries of origin, Extent of Occurrence (EOO), individual PAs where the species is recorded and known threats. We obtained spatial data on PAs from the World Database of Protected Areas (WDPA) administered by UNEP-World Conservation Monitoring Centre [38], which was comprised of 84488 protected area polygons.

We downloaded CR frog distribution data from the IUCN Red List for the ‘Amphibian-Anuran’ category on 6 July 2011. These data depicted the spatial extent of occurrence (EOO) for all CR frog species at a global scale. To determine the extent of CR frog distributions by country and within PAs we overlaid the frog data with the WDPA layer to select those PAs intersected by the EOO of any CR frog species. We cleaned the resultant subset of PAs to remove duplicates and topological data errors. Such anomalies arise due to areas being captured from multiple data sources (e.g. WCMC database, UNESCO World Heritage Site database, UNESCO Man and Biosphere database, etc.). This refinement was necessary to avoid duplication which would have overestimated the proportions of CR frogs conserved. Our final database contains 1152 PAs each overlapping with the EOO of at least one CR frog species.

Once cleaned and consolidated, we projected both CR frog and PA spatial layers using the Eckert IV global projection, WGS 1984 datum. We calculated the EOO area for each frog species and the area of each PA. New area calculations provided a consistent benchmark for frogs and PAs at a global scale. We completed the spatial geoprocessing of the data in ESRI ArcGIS 9.3. Congruence between CR frog and PA data layers was determined as follows. First, we used the ‘union’ tool in ArcGIS to produce a series of unique polygons for each species. The ‘union’ function computes the geometric intersection of multiple polygon features, in this case frog EOO and PA boundaries, such that the resultant output comprises all combinations of frog species and PAs. We then exported this new feature database to Excel where we used pivot tables to determine the following summary data for each frog species and PA: (i) a list of frog species recorded within PAs, (ii) extent of each frog species EOO in each country, (iii) extent of each frog species EOO within PAs for each country, (iv) the number of frog species protected in multiple PAs, and their extent in each.

### Financial contribution of tourism to PA budgets

We extracted data on budgets and revenue sources (e.g. government, NGOs, donations, tourism) for each relevant protected area agency from: the agencies' annual reports, operating reports and in some cases, financial statements for individual PAs. Published reports are available for some countries [19]. For others, we contacted management agencies directly to obtain budget data. In most cases we were able to obtain income and expenditure and/or cash flow statements specifying income sources. We were thus able to determine the proportion of funding generated from tourism activities through mechanisms such as visitor entry fees, donations, accommodation and concessions.

We were unable to determine the proportion of tourism funding for individual PAs as these figures are frequently only reported at an agency level. Consequently we use a national level approach to permit comparisons between species, countries and/or regions. We were also unable to determine the exact nature of the activities supported by tourism funding in each PA as this information is not available for the vast majority of PAs.

### The overall contribution of tourism to CR frog conservation

We used a simple accounting approach to quantify the contribution of tourism, *T*, to the protection of each CR frog species based on Equation 1 below:
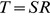
(1)Where *S* = the proportion of each species' geographic range occurring in PAs and *R* = proportions of operating revenue which are derived from tourism for those PAs. By quantifying the proportion of each species effectively protected by tourism, *T* provides an indication of the relative importance of tourism revenue in CR frog conservation. This approach implicitly assumes that individuals are distributed evenly across their entire range. Whilst rarely accurate, it is the least biased assumption that can be made without more detailed data on species distributions.

Using data at the global scale allows us to make comparisons between species, funding sources and regions without the associated confounding local political, financial and conservation management factors.

## Results

### Summary of CR frogs

There are 415 CR frog species in total found in 52 countries globally (Table 1, Table S1). The majority of CR species are found in the Neotropics (318 species, 11% of all anurans in region), followed by the Afrotropical and Indomalayan regions (34 species, 3% each). There are few CR frogs in the Australasian (18 species, 4%), Palearctic (9, 2%) and Nearctic (2, 2%) regions.

**Table 1:** Summary of the number of Critically Endangered (CR) frog species, the number of CR species found in a Protected Area (PA), the average proportion of CR species ranges occurring in PAs, and the average proportion of CR species ranges protected by tourism in each country.

Region^a^	Country	Number of CR species	Number of CR species in PA	Mean (± 1SD) proportion of each species range in PA (%)	Mean (± 1SD) proportion of ranges protected by tourism (%)^b^
Afrotropical	Cameroon	7	5	38.8±38.1	?
Afrotropical	Congo	1	1	100±0.0	?
Afrotropical	Côte d'Ivoire	2	2	79.7±17.7	?
Afrotropical	Gabon	1	0	0±0.0	0±0.0
Afrotropical	Ghana	2	2	2.5±1.1	?
Afrotropical	Guinea	1	1	91±0.0	?
Afrotropical	Kenya	1	1	33.2±0.0	43.4±0.0
Afrotropical	Madagascar	7	4	19.4±37.0	1.7±1.8
Afrotropical	South Africa	5	4	24.4±35.5	14.4±16.7
Afrotropical	Tanzania	6	6	72.8±29.5	26.7±10.8
Afrotropical	Togo	1	1	1.0±0.0	?
Afrotropical	Uganda	1	1	33.2±0.0	?
Afrotropical	Zimbabwe	1	1	98.5±0.0	98.5±0.0
Australasian	Australia	15	15	42.3±25.0	3.9±2.2
Australasian	New Zealand	1	1	53.3±0.0	4.2±0.0
Australasian	Papua New Guinea	1	0	0±0.0	0±0.0
Indomalayan	India	13	5	15.7±37.4	4.1±3.0
Indomalayan	Indonesia	3	2	12.5±11.2	?
Indomalayan	Malaysia	3	3	29.7±3.8	?
Indomalayan	Philippines	1	0	0±0.0	0±0.0
Indomalayan	Sri Lanka	11	4	19.0±40.1	?
Nearctic	United States	2	2	18.7±18.8	1.4±1.4
Neotropic	Argentina	2	2	100±0.0	26.5±0.0
Neotropic	Bolivia	10	7	51.9±43.3	6.2±3.5
Neotropic	Brazil	9	2	21.0±41.8	7.4±3.3
Neotropic	British Virgin Islands	1	1	3.0±0.0	1.1±0.0
Neotropic	Chile	9	4	7.2±15.5	6.1±5.9
Neotropic	Colombia	52	26	30.2±41.1	4.9±3.1
Neotropic	Costa Rica	19	18	45.5±19.9	7.6±3.3
Neotropic	Cuba	16	15	54.4±36.6	3.1±1.8
Neotropic	Dominican Republic	11	8	10.2±14.9	2.2±2.4
Neotropic	Ecuador	35	18	15.7±22.6	6.9±6.2
Neotropic	El Salvador	1	1	9.3±0.0	0.65±0.0
Neotropic	Guatemala	21	15	19±25.7	10.4±5.7
Neotropic	Haiti	31	0	0±0.0	0±0.0
Neotropic	Honduras	23	18	49.9±34.3	14.9±8.6
Neotropic	Jamaica	7	7	60.6±37.1	?
Neotropic	Mexico	43	11	13.3±26.6	3.9±1.6
Neotropic	Montserrat	1	0	0±0.0	0±0.0
Neotropic	Nicaragua	2	2	29.6±11.1	3.2±0.0
Neotropic	Panama	22	20	43.4±39.5	6.8±5.2
Neotropic	Peru	23	5	8.6±23.1	6.6±3.6
Neotropic	Puerto Rico	7	6	11.5±8.1	?
Neotropic	Trinidad and Tobago	2	2	45.3±14.7	?
Neotropic	Uruguay	1	0	0±0.0	0±0.0
Neotropic	Venezuela	18	17	69.8±28.1	9.2±3.5
Palearctic	China	5	3	14.4±26.7	?
Palearctic	Greece	1	0	0±0.0	0±0.0
Palearctic	Israel	1	1	0.3±0.0	?
Palearctic	Liberia	1	1	100±0.0	?
Palearctic	Palestine	1	0	0±0.0	0±0.0
Palearctic	Turkey	1	0	0±0.0	0±0.0

Details for individual species can be found in Table S1.

a Regions based on [52].

b ? = financial data unavailable for country.

All 415 species are threatened by multiple processes. Habitat loss, including fragmentation or modification, is the most important single threat affecting all CR frog species. Invasive species threaten 214 CR frog species and pollution threatens 116 species. Overexploitation through direct harvesting threatens 21 species. Compounding or exacerbating these threats are the small geographic ranges of most species (274 species).

### Protected areas

**Figure 1:**
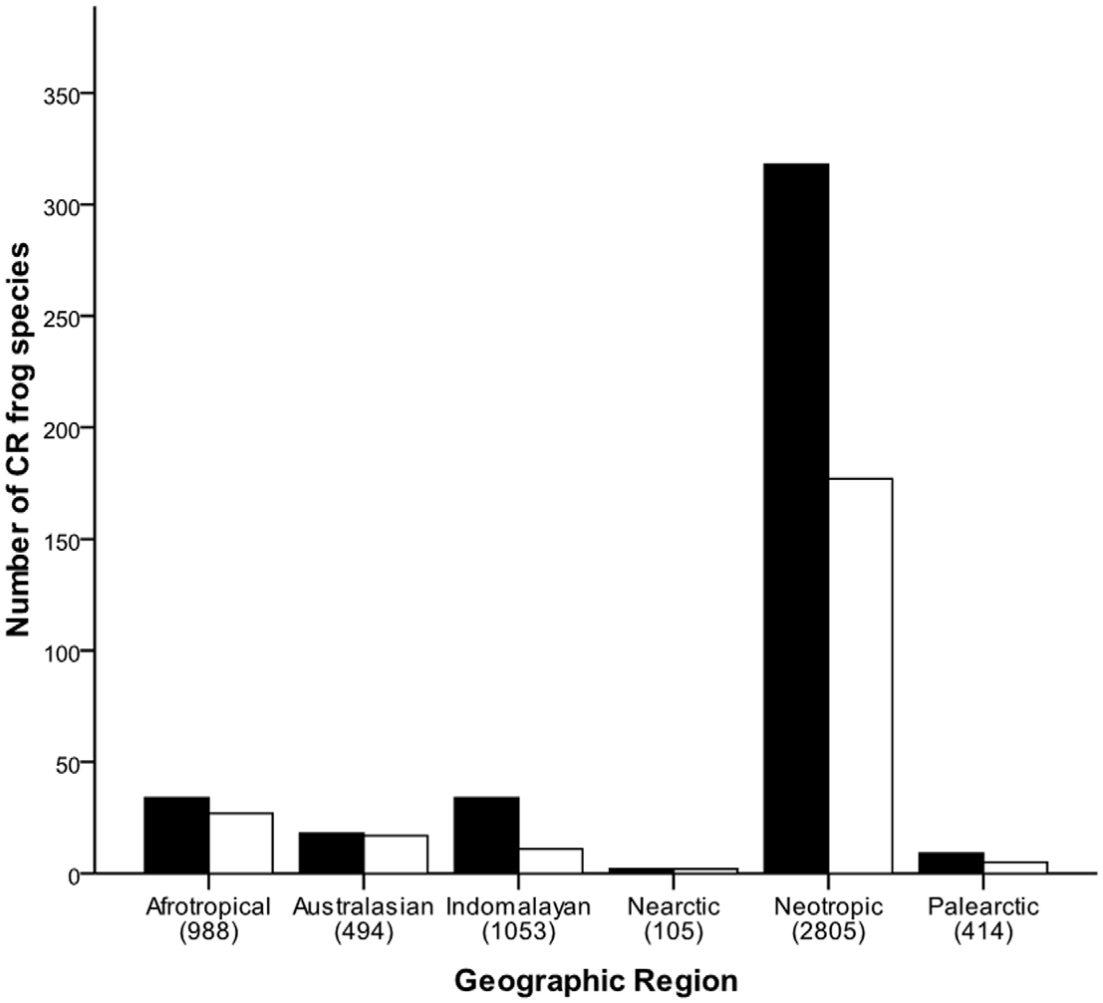
Total number of CR frog species (black bars) and CR frog species in protected areas (white bars) in each geographic region. Numbers in parentheses represent the total number of frog species in each region.

Roughly 60% (239 species) of CR frogs have all or part of their geographic range within one or more PA (Table 1, Table S1). The remaining 176 species do not occur in any PA. More species are found within PAs in the Neartic (100%), Australasian (94%) and Afrotropical (79%) regions (Fig. 1). Proportionately fewer species in the Neotropical (56%), Palearctic (56%) and Indomalayan (32%) regions are found in PAs.

**Figure 2:**
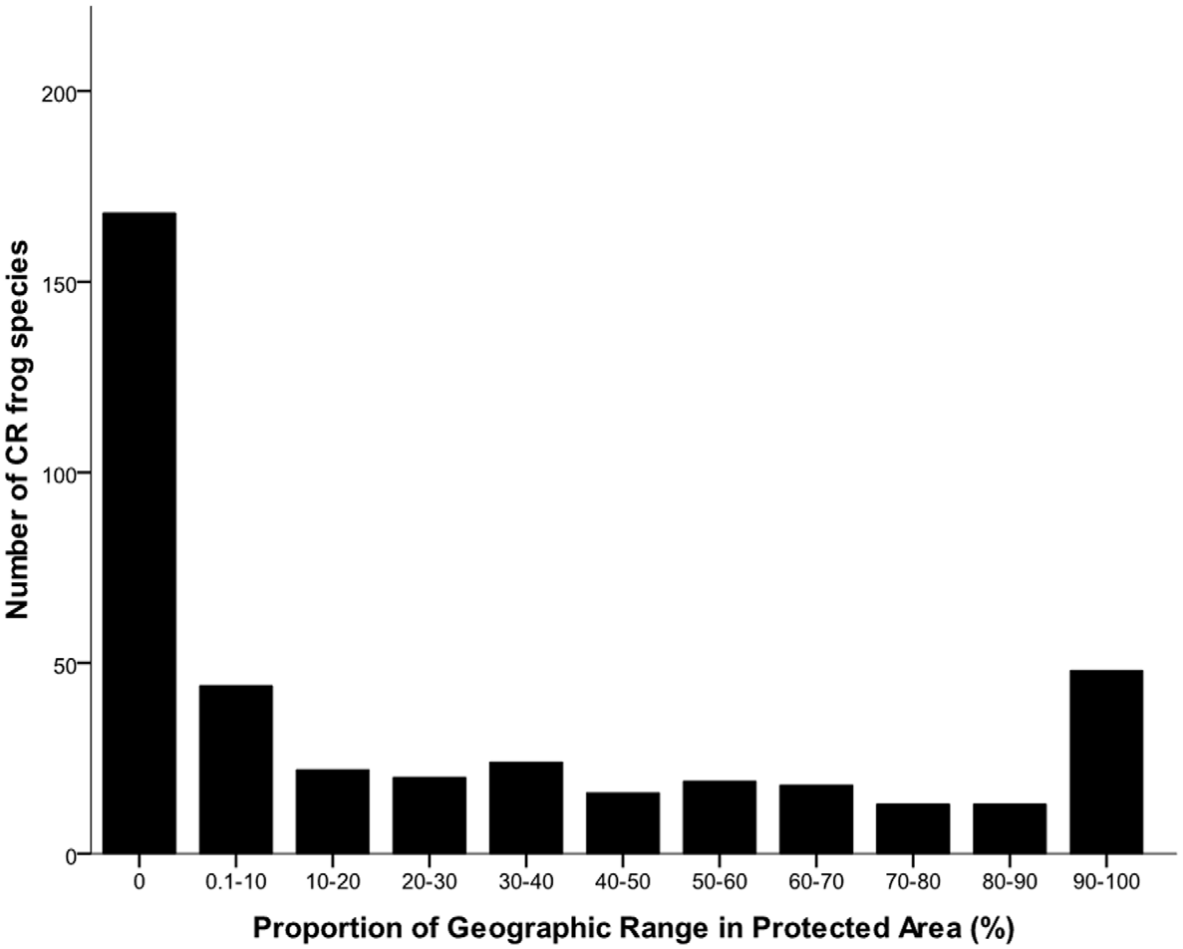
Proportion of CR frog species geographic ranges covered by protected area systems.

**Figure 3:**
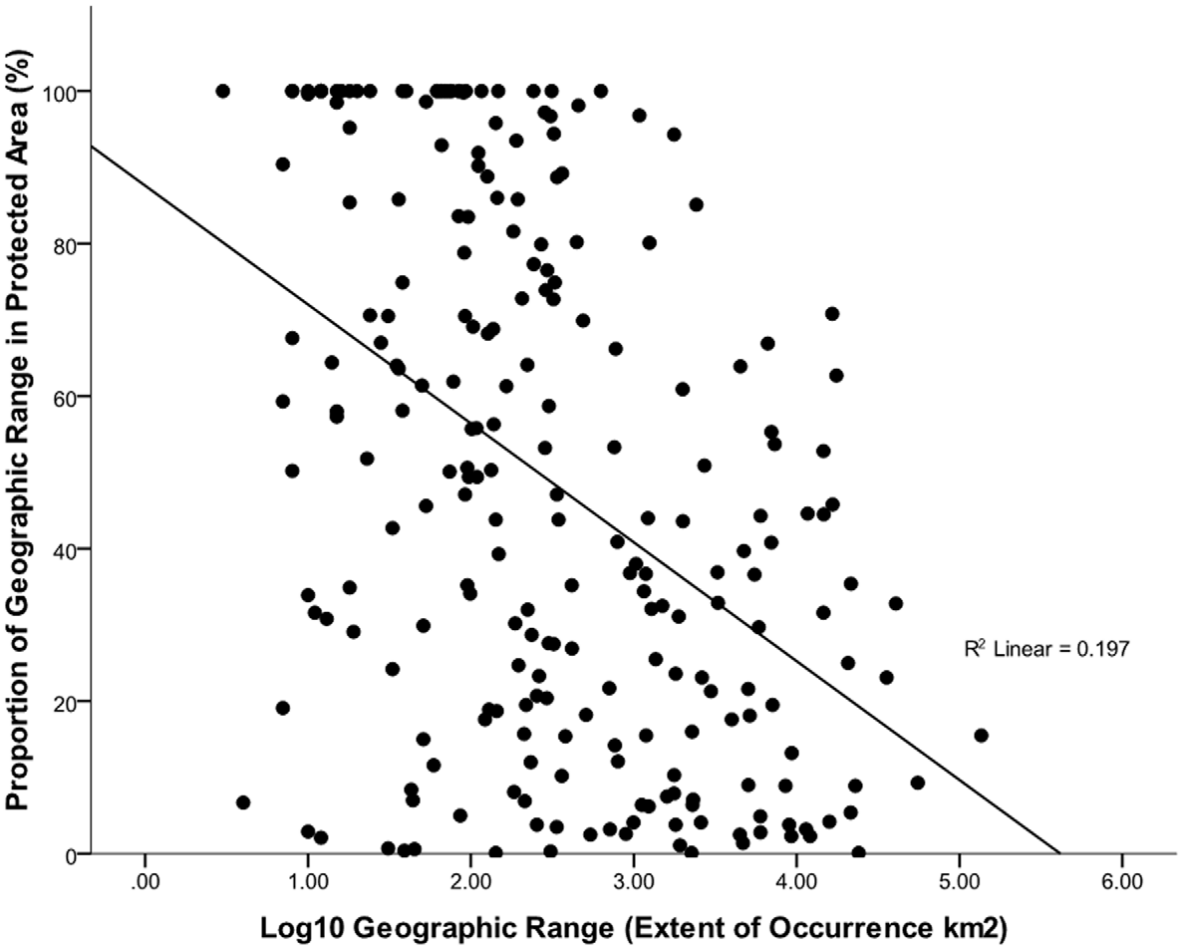
Relationship between geographic range size and proportion of range in protected area (only for species found in at least 1 PA).

Of the protected species, the proportion of their geographic range occurring in a PA ranges from 0.3–100% (Fig. 2). The average proportion of species geographic ranges covered by PA systems is highest in Afrotopical (45%) and Australasian (40%) regions (Table 1). Proportionately less of each species geographic range is protected in the Neotropical (28%), Palearctic (19%), Nearctic (19%), and Indomalayan (16%) regions.

he proportion of species ranges occurring in PAs is not an artefact of species range size when all species were considered (R^2^ = 0.0011, n = 415, p>0.05). If only considering species found in PAs, we found a negative relationship between geographic range size and proportion of range in PAs, i.e. the smaller the geographic range, the higher the proportion of the range within a PA (R^2^ = 0.04, n = 239, p = 0.002, Fig. 3).

### Tourism contribution to CR frog protection

Critically endangered frogs are recorded from 52 countries. We were able to obtain financial information for 33 countries (65%). For these countries, the proportion of PA budgets derived from tourism range from 5–100% (Table 2, Table S2). Tourism contributions to PAs are highest in the Afrotropical (51%), Indomalayan (30%) and Neotropical (18%) regions. Less than 10% of PA budgets were derived from tourism in the Australasian (9%) and Nearctic (7%) regions which were primarily funded by government contributions. No data are available on the contribution of tourism to PAs in the Palearctic region.

**Table 2:** Contribution of tourism [R] to protected area (PA) management budgets in each country where data is available (see Table S2 for details).

Country	Proportion of PA budget from tourism [R]	Country	Proportion of PA budget from tourism [R]
Argentina	26.5	Kenya	66.1
Australia	9.4	Madagascar	5.0
Bolivia	8.1	Mexico	5.9
Brazil	7.8	New Zealand	7.9
British Virgin Islands	58.2	Nicaragua	8.3
Chile	37.9	Panama	13.1
Colombia	7.6	Peru	15.5
Costa Rica	18.2	Philippines	53.0
Cuba	5.0	South Africa	47.2
Dominican Republic	15.8	Tanzania	36.7
Ecuador	27.6	United States	7.4
El Salvador	6.9	Uruguay	8.1
Guatemala	30.8	Venezuela	12.4
Honduras	25.0	Zimbabwe	100.0

Of the 415 CR frog species, 353 occur in the 33 countries where PA financial data are available. Geographic ranges for 201 (58%) of these species overlap with at least one PA (Table S1)

The 19 countries where data on the financial contribution of tourism to PA management budgets are not available are: Cameroon, China, Congo, Côte d'Ivoire, Gabon, Ghana, Greece, Haiti, Indonesia, Israel, Jamaica, Liberia, Malaysia, Montserrat, Puerto Rico, Sri Lanka, Togo, Trinidad and Tobago, and Turkey. These countries support populations of 62 CR frog species, of which 61 occur in at least one PA. This suggests that the total number of species protected by tourism is well above the minimum of 201 calculated above.

**Figure 4:**
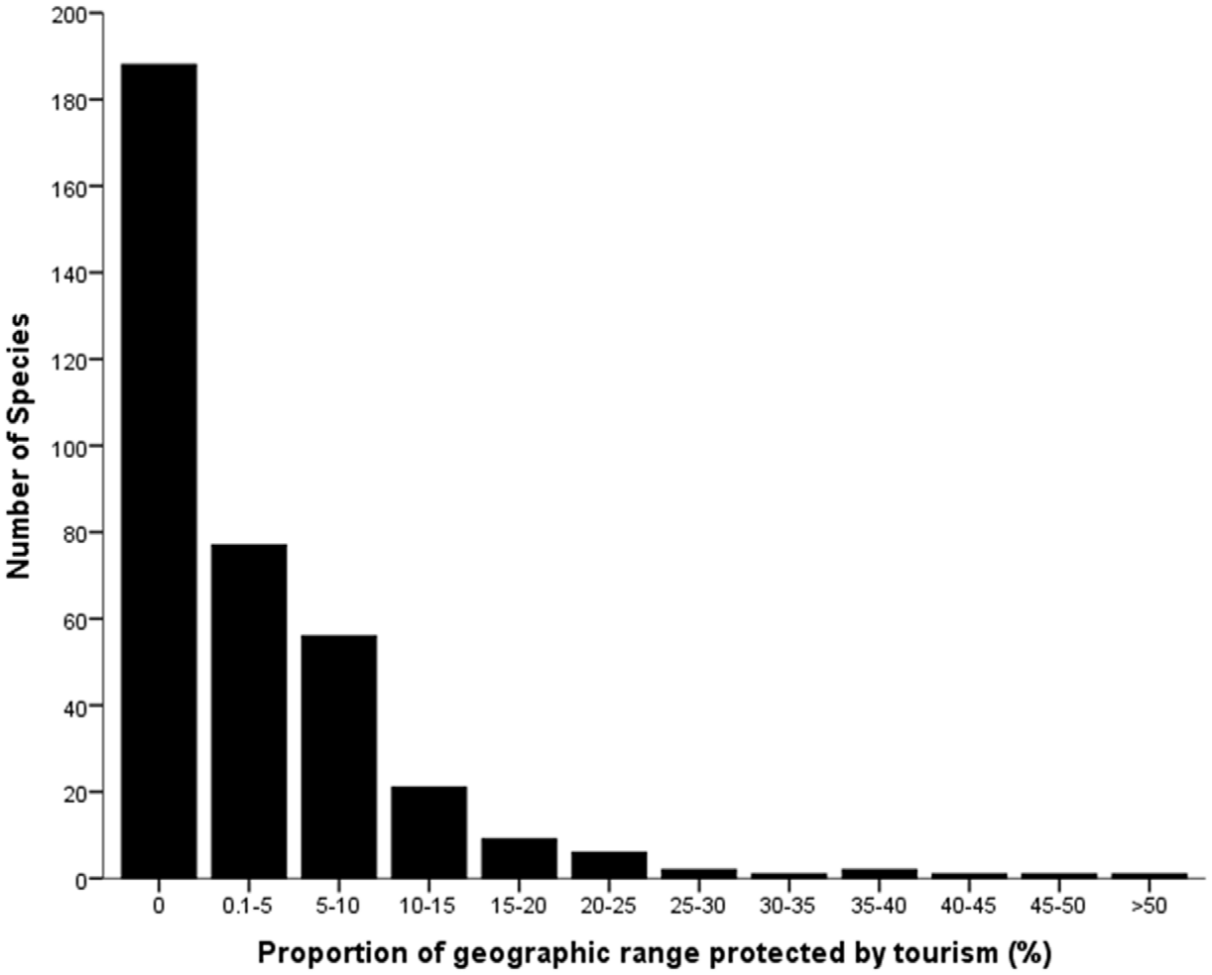
Proportion [T] of CR frog species geographic ranges for which conservation funding is derived from tourism.

The proportion of individual CR frog species global geographic ranges for which conservation funding is derived from tourism ranges from 0–99% (Table 1, Fig. 4, Table S1). The overall contribution of tourism to CR frog species protection was important (>5%, *sensu* [39]) for 115 species with a further 71 species receiving protection for 0.5–5% of their global populations through tourism. The overall proportion of each species protected by tourism was highest in the Afrotropical region (average of 18%) which had the highest average contribution of tourism (98.5%) and >30% of CR species in protected areas. Despite high protection levels, the lowest overall proportion of each species protected by tourism was in the developed Australasian (4%) and Nearctic (2%) regions with <10% of protected area budgets funded by tourism. Low average tourism contribution to critically endangered frog species protection in the Neotropical (7%) and Indomalayan (4%), regions is a combination of low species coverage in PAs and low tourism contributions to PA budgets.

### Negative impacts of tourism on frogs

Tourism is recorded as generating negative impacts on 65 CR frog species (15.7%) in 21 different countries [37] (Table S2). Most of these species are found in the Neotropical (38 species), Australasian (10) and Indomalayan (10) regions. Few species are known to be threatened by tourism in the Afrotropical (3) and Palaearctic (3) regions and none are listed as threatened by tourism in the Nearctic region.

The principal processes by which tourism constitutes a threat to CR frog species are: habitat destruction or modification by tourism infrastructure development and/or construction (19 species); recreational activities including hiking and watersports (27 species); or both (19 species). More than half of these 65 species are moderately (18 species) or severely (17 species) threatened by tourism (Table S3). Moderate and severe tourism impacts are more common in the Indomalayan (70% of regional species) and Neotropical (62%) regions and less common in the Afrotopical (34%) and Australasian (9%) regions. Forty-two of the 65 CR frog species threatened by tourism also receive direct conservation funding from tourism.

## Discussion

### Conservation of CR frogs in PAs

Protected areas are important for CR frog conservation with 60% of CR frog species occurring in PAs. It is not possible, however, to accurately determine how effective PAs are in safeguarding these species due to data limitations for frog species life histories, distributions and conservation management practices. Data on amphibian species distributions are generally very coarse. For many species the only distribution maps show generalized Extent of Occurrence (EOO) which may include large areas of unsuitable habitat rather than Area of Occupancy (AOO) showing where these frogs are actually known to occur [40], [41]. Records of frogs found in individual PAs are sometimes also incomplete or erroneous. Some PAs may also offer very limited protection in practice, though even ‘paper parks’ may be better than nothing at all [42]. It is also important to note that the presence of a species within a PA may not influence its status as the PA may not protect it from all threatening processes [43].

Proportionately fewer threatened amphibians (73%) are found in PAs than for threatened mammals (86%), birds (80%), and turtles (90%) [4]. There are several possible reasons: (i) most amphibians are associated with freshwater habitats which are poorly represented by terrestrial PA networks [42], [44], (ii) few protected areas were established specifically to capture amphibian distributions [9], (iii) on average, amphibians have smaller ranges than mammals and birds so incidental occurrence in PAs is less likely [9]. Our results show no correlation between overall range size for individual CR frogs and the proportions of range occurring within PAs. If only those species that actually do occur in PAs are considered however, the proportions of ranges protected are inversely related to overall range area. Thus, the smaller the range size, the higher the proportion of the range within a protected area. This suggests that most CR frogs with very small ranges (EOO<10 km^2^) are likely to be included in PA systems.

CR frog coverage in PAs was lowest in the Neotropical (56%) and Indomalayan (32%) regions, particularly in Haiti (30 CR species, 0 in PAs). Similar studies have reported the major gaps in PA coverage for amphibians in low-income tropical countries, particularly in tropical and subtropical moist forest habitats and on tropical islands [9]. While the proportion of CR frog species not protected in the Palearctic region was also relatively high (60%) in our study, this only represents 6 species.

### Tourism contributions to CR frog protection

Tourism focused specifically on frogs is much less common or popular than that focused on mammals or birds [45], [46], [47]. Frogs are however, often used to advertise more generic forms of ecotourism or nature-based tourism, e.g. in logos such as that of the Rainforest Alliance. This indicates a broad appeal which may potentially attract attention from conservation finance organizations. Most unprotected CR frog species occur in low-income tropical countries, which can least afford the costs of establishing and managing protected areas [12], [18]. Furthermore, few PAs were established and/or managed specifically for frog conservation and/or frog-related tourism. The main benefit to frogs from tourism therefore is indirect, from the establishment and funding of PAs for biodiversity protection more generally or in scenically attractive areas.

Despite not being focal species for nature-based tourism, our results indicate that tourism contributes to the protection of more than half of CR frog species. Relatively more of these species are protected by tourism in the Afrotropical, Neotropical and Indomalayan regions, areas with high tourism contributions to PA budgets and relatively high numbers of CR species in PAs. The CR frog species most at risk are those whose conservation is not funded through tourism, or any other mechanism, and whose distribution does not overlap with any PAs (176 species).

### Enhancing tourism revenue to protected areas

Our study and others [19], [20], [39], [48], reveal that tourism represents a significant proportion (>30%) of PA budgets in some countries, particularly in developing regions, e.g. Zimbabwe, Kenya, Tanzania, British Virgin Islands, and Chile. Developing countries can potentially increase the contribution of tourism to PAs by introducing entrance or access fees, or by increasing current fees, an approach justified by studies demonstrating tourist willingness to pay higher access fees in some countries [30], [35], [49]. In these cases PA management will have to make decisions regarding tradeoffs between the time and resources spent managing tourists and/or tourism infrastructure and their conservation activities.

While our study highlights the significant contributions that tourism can make to conservation efforts, it is important that PA agencies do not rely solely on tourism as the primary source of income. Fluctuations in the tourism market resulting from events such as the Global Financial Crisis and political instability will have significant flow-on effects for tourism-generated PA revenue. For example, visits to National Parks in Kenya, Uganda and Zimbabwe, declined markedly during periods of civil unrest in the 1990s. In Zimbabwe prior to 1999, more than 90% of the PA budget was derived from over 1.5 million tourist arrivals/year [50]. Civil unrest and economic instability prevalent in 1999 and 2000 saw visitor arrivals drop by 70% until 2006 with a corresponding decrease in PA tourism derived-funding [50], [51]. Despite these trends Zimbabwe still funds conservation efforts in PAs entirely through tourism revenues. In contrast, developed countries such as Australia, New Zealand and the United States are much less reliant on tourism revenue (<10% of PA budgets) due to high government contributions and are therefore more resilient to tourism fluctuations. Consequently, less developed countries need to consider alternative funding mechanisms (e.g. payments for ecosystem services, carbon offset schemes, etc.) to diversify income streams while also supplementing and enhancing tourism revenue.

## Supporting Information

Table S1 **This table presents a summary of all 415 CR frogs, their geographic location, range size and proportion of their population (range) protected by tourism.**
(DOC)

Table S2 **This table presents the financial contributions to protected areas from different funding sources in each country where data are available.**
(DOC)

Table S3 **This table presents the CR frog species threatened by tourism activities.**
(DOC)
