# Supplementary material for: Tourism and the Conservation of Critically Endangered Frogs
Source: PLoS One. 2012 Sep 12;7(9):e43757. doi: 10.1371/journal.pone.0043757 (PMC3440435; doi:10.1371/journal.pone.0043757)
Supplement: Table S1 — This table presents a summary of all 415 CR frogs, their geographic location, range size and proportion of their population (range) protected by tourism. (DOC) [file pone.0043757.s001.doc]

**Table S1**. Summary of CR frogs, geographic location, range size and proportion of range protected by tourism. N/A = data not available.

| **Scientific Name** | **Region** | **Countries** | **Extent of Occurrence EOO (km^2^)** | **EOO (km^2^) occurring in PAs** | **Tourism contribution to PA budget (%)** | **Proportion of range protected by tourism (%)** | **Tourism a threat ?** |
| --- | --- | --- | --- | --- | --- | --- | --- |
| *Adenomus dasi* | Indomalayan | Sri Lanka | 39 | 0.1 | N/A | N/A | N |
| *Agalychnis moreletii* | Neotropical | Belize, El Salvador, Guatemala, Honduras, Mexico | 35672 | 8241.2 | 15.6 | 3.70 | N |
| *Albericus siegfriedi* | Australasian | Papua New Guinea | 17 | 0.0 | 0.0 | 0.00 | N |
| *Alexteroon jynx* | Afrotropical | Cameroon | 218 | 42.5 | N/A | N/A | N |
| *Allobates juanii* | Neotropical | Colombia | 69 | 0.0 | 7.6 | 0.00 | N |
| *Alsodes montanus* | Neotropical | Chile | 22 | 0.0 | 37.9 | 0.00 | Y |
| *Alsodes tumultuosus* | Neotropical | Chile | 52 | 0.0 | 37.9 | 0.00 | Y |
| *Alsodes vanzolinii* | Neotropical | Chile | 9 | 0.0 | 37.9 | 0.00 | N |
| *Ameerega ingeri* | Neotropical | Colombia | 16 | 0.0 | 7.6 | 0.00 | N |
| *Ameerega planipaleae* | Neotropical | Peru | 222 | 142.6 | 15.5 | 9.94 | N |
| *Amietophrynus taiensis* | Afrotropical | Côte d'Ivoire | 6651 | 4450.6 | N/A | N/A | N |
| *Andinophryne colomai* | Neotropical | Ecuador | 75 | 0.0 | 27.6 | 0.00 | N |
| *Aromobates leopardalis* | Neotropical | Venezuela | 38 | 38.0 | 12.4 | 12.40 | N |
| *Aromobates meridensis* | Neotropical | Venezuela | 206 | 149.7 | 12.4 | 9.03 | N |
| *Aromobates nocturnus* | Neotropical | Venezuela | 187 | 56.5 | 12.4 | 3.75 | N |
| *Arthroleptella rugosa* | Afrotropical | South Africa | 7 | 0.0 | 47.2 | 0.00 | N |
| *Arthroleptis troglodytes* | Afrotropical | Zimbabwe | 15 | 14.6 | 100.0 | 98.5 | N |
| *Astylosternus nganhanus* | Afrotropical | Cameroon | 170 | 0.0 | N/A | N/A | N |
| *Atelopus andinus* | Neotropical | Peru | 2420 | 2058.4 | 15.5 | 13.18 | N |
| *Atelopus angelito* | Neotropical | Colombia | 69 | 0.0 | 7.6 | 0.00 | N |
| *Atelopus arsyecue* | Neotropical | Colombia | 74 | 73.7 | 7.6 | 7.60 | N |
| *Atelopus arthuri* | Neotropical | Ecuador | 74 | 0.0 | 27.6 | 0.00 | N |
| *Atelopus balios* | Neotropical | Ecuador | 383 | 0.0 | 27.6 | 0.00 | N |
| *Atelopus bomolochos* | Neotropical | Ecuador | 11419 | 369.8 | 27.6 | 0.89 | N |
| *Atelopus boulengeri* | Neotropical | Ecuador | 2399 | 0.0 | 27.6 | 0.00 | N |
| *Atelopus carauta* | Neotropical | Colombia | 142 | 0.1 | 7.6 | 0.01 | N |
| *Atelopus carbonerensis* | Neotropical | Venezuela | 145 | 124.7 | 12.4 | 10.67 | N |
| *Atelopus carrikeri* | Neotropical | Colombia | 628 | 628.2 | 7.6 | 7.60 | N |
| *Atelopus chiriquiensis* | Neotropical | Costa Rica, Panama | 4514 | 2883.1 | 16.4 | 10.22 | Y |
| *Atelopus chocoensis* | Neotropical | Colombia | 345 | 0.0 | 7.6 | 0.00 | N |
| *Atelopus chrysocorallus* | Neotropical | Venezuela | 66 | 66.4 | 12.4 | 12.40 | N |
| *Atelopus coynei* | Neotropical | Ecuador | 2975 | 634.0 | 27.6 | 5.88 | N |
| *Atelopus cruciger* | Neotropical | Venezuela | 6995 | 3868.0 | 12.4 | 6.86 | N |
| *Atelopus ebenoides* | Neotropical | Colombia | 1921 | 20.6 | 7.6 | 0.08 | N |
| *Atelopus elegans* | Neotropical | Colombia, Ecuador | 9266 | 214.2 | 7.6 | 0.18 | N |
| *Atelopus epikeisthos* | Neotropical | Peru | 30 | 0.0 | 15.5 | 0.00 | N |
| *Atelopus erythropus* | Neotropical | Peru | 267 | 0.0 | 15.5 | 0.00 | N |
| *Atelopus eusebianus* | Neotropical | Colombia | 732 | 0.0 | 7.6 | 0.00 | N |
| *Atelopus exiguus* | Neotropical | Ecuador | 765 | 108.9 | 27.6 | 3.93 | N |
| *Atelopus famelicus* | Neotropical | Colombia | 233 | 27.9 | 7.6 | 0.91 | N |
| *Atelopus farci* | Neotropical | Colombia | 100 | 0.0 | 7.6 | 0.00 | N |
| *Atelopus galactogaster* | Neotropical | Colombia | 65 | 64.5 | 7.6 | 7.60 | N |
| *Atelopus glyphus* | Neotropical | Colombia, Panama | 338 | 299.4 | 13.1 | 11.53 | N |
| *Atelopus guanujo* | Neotropical | Ecuador | N/A | N/A | 27.6 | N/A | N |
| *Atelopus guitarraensis* | Neotropical | Colombia | 66 | 0.0 | 7.6 | 0.00 | N |
| *Atelopus halihelos* | Neotropical | Ecuador | 22 | 0.0 | 27.6 | 0.00 | N |
| *Atelopus laetissimus* | Neotropical | Colombia | 147 | 146.8 | 7.6 | 7.60 | Y |
| *Atelopus lozanoi* | Neotropical | Colombia | 446 | 357.8 | 7.6 | 6.09 | N |
| *Atelopus lynchi* | Neotropical | Ecuador | 42 | 0.0 | 27.6 | 0.00 | N |
| *Atelopus mandingues* | Neotropical | Colombia | 288 | 213.3 | 7.6 | 5.62 | N |
| *Atelopus mindoensis* | Neotropical | Ecuador | 3993 | 701.4 | 27.6 | 4.85 | N |
| *Atelopus minutulus* | Neotropical | Colombia | 24 | 0.0 | 7.6 | 0.00 | N |
| *Atelopus monohernandezii* | Neotropical | Colombia | 102 | 0.0 | 7.6 | 0.00 | N |
| *Atelopus mucubajiensis* | Neotropical | Venezuela | 90 | 89.8 | 12.4 | 12.38 | Y |
| *Atelopus muisca* | Neotropical | Colombia | 137 | 94.4 | 7.6 | 5.23 | N |
| *Atelopus nahumae* | Neotropical | Colombia | 284 | 275.7 | 7.6 | 7.39 | N |
| *Atelopus nanay* | Neotropical | Ecuador | 8 | 5.6 | 27.6 | 18.66 | N |
| *Atelopus nepiozomus* | Neotropical | Ecuador | 2277 | 363.5 | 27.6 | 4.41 | N |
| *Atelopus nicefori* | Neotropical | Colombia | 91 | 71.7 | 7.6 | 5.99 | N |
| *Atelopus onorei* | Neotropical | Ecuador | 2 | 0.0 | 27.6 | 0.00 | N |
| *Atelopus oxyrhynchus* | Neotropical | Venezuela | 345 | 150.9 | 12.4 | 5.43 | N |
| *Atelopus pachydermus* | Neotropical | Colombia, Ecuador, Peru | 8987 | 338.0 | 27.1 | 1.02 | N |
| *Atelopus patazensis* | Neotropical | Peru | N/A | N/A | 15.5 | N/A | N |
| *Atelopus pedimarmoratus* | Neotropical | Colombia | 97 | 0.0 | 7.6 | 0.00 | N |
| *Atelopus peruensis* | Neotropical | Peru | 22915 | 2047.6 | 15.5 | 1.39 | N |
| *Atelopus petersi* | Neotropical | Ecuador | 7332 | 3938.3 | 27.6 | 14.82 | N |
| *Atelopus petriruizi* | Neotropical | Colombia | 313 | 313.1 | 7.6 | 7.60 | N |
| *Atelopus pictiventris* | Neotropical | Colombia | 301 | 176.4 | 7.6 | 4.46 | N |
| *Atelopus pinangoi* | Neotropical | Venezuela | 47 | 0.0 | 12.4 | 0.00 | N |
| *Atelopus planispina* | Neotropical | Ecuador | 3263 | 1204.4 | 27.6 | 10.19 | N |
| *Atelopus pulcher* | Neotropical | Peru | 9320 | 1232.2 | 15.5 | 2.05 | N |
| *Atelopus pyrodactylus* | Neotropical | Peru | 24 | 0.0 | 15.5 | 0.00 | N |
| *Atelopus quimbaya* | Neotropical | Colombia | 254 | 52.7 | 7.6 | 1.57 | N |
| *Atelopus reticulatus* | Neotropical | Peru | 68 | 0.0 | 15.5 | 0.00 | N |
| *Atelopus seminiferus* | Neotropical | Peru | 2278 | 0.0 | 15.5 | 0.00 | N |
| *Atelopus senex* | Neotropical | Costa Rica | 1154 | 397.6 | 18.2 | 6.27 | N |
| *Atelopus sernai* | Neotropical | Colombia | 310 | 0.0 | 7.6 | 0.00 | N |
| *Atelopus simulatus* | Neotropical | Colombia | 353 | 0.0 | 7.6 | 0.00 | N |
| *Atelopus sonsonensis* | Neotropical | Colombia | 355 | 0.0 | 7.6 | 0.00 | N |
| *Atelopus sorianoi* | Neotropical | Venezuela | 182 | 148.8 | 12.4 | 10.12 | N |
| *Atelopus subornatus* | Neotropical | Colombia | 308 | 0.9 | 7.6 | 0.02 | N |
| *Atelopus tamaense* | Neotropical | Colombia, Venezuela | 66 | 61.5 | 10.2 | 9.29 | N |
| *Atelopus varius* | Neotropical | Costa Rica, Panama | 21540 | 7620.2 | 16.9 | 6.01 | N |
| *Atelopus walkeri* | Neotropical | Colombia | 190 | 177.8 | 7.6 | 7.10 | N |
| *Atelopus zeteki* | Neotropical | Panama | 1599 | 120.0 | 13.1 | 0.98 | Y |
| *Atopophrynus syntomopus* | Neotropical | Colombia | 6 | 0.0 | 7.6 | 0.00 | N |
| *Bokermannohyla izecksohni* | Neotropical | Brazil | 66 | 0.0 | 7.8 | 0.00 | N |
| *Boophis williamsi* | Afrotropical | Madagascar | 366 | 0.0 | 5.0 | 0.00 | N |
| *Bromeliohyla dendroscarta* | Neotropical | Mexico | 1839 | 0.0 | 5.9 | 0.00 | N |
| *Cardioglossa alsco* | Afrotropical | Cameroon | 62 | 61.5 | N/A | N/A | N |
| *Cardioglossa trifasciata* | Afrotropical | Cameroon | 15 | 8.3 | N/A | N/A | N |
| *Centrolene ballux* | Neotropical | Colombia, Ecuador | 237 | 0.0 | 7.6 | 0.00 | N |
| *Centrolene gemmatum* | Neotropical | Ecuador | 38 | 22.0 | 27.6 | 16.03 | N |
| *Centrolene heloderma* | Neotropical | Colombia, Ecuador | 5151 | 933.6 | 7.6 | 1.38 | N |
| *Charadrahyla altipotens* | Neotropical | Mexico | 11 | 0.0 | 5.9 | 0.00 | N |
| *Charadrahyla trux* | Neotropical | Mexico | 80 | 0.0 | 5.9 | 0.00 | N |
| *Churamiti maridadi* | Afrotropical | Tanzania | 95 | 48.3 | 36.7 | 18.57 | N |
| *Colostethus jacobuspetersi* | Neotropical | Ecuador | 5050 | 453.9 | 27.6 | 2.48 | N |
| *Conraua derooi* | Afrotropical | Ghana, Togo | 4478 | 112.8 | N/A | N/A | N |
| *Cophixalus concinnus* | Australasian | Australia | 14 | 9.1 | 9.4 | 6.05 | Y |
| *Cophyla berara* | Afrotropical | Madagascar | 10 | 9.6 | 5.0 | 4.98 | N |
| *Craugastor anciano* | Neotropical | Honduras | 95 | 33.3 | 25.0 | 8.81 | N |
| *Craugastor andi* | Neotropical | Costa Rica | 2001 | 871.9 | 18.2 | 7.93 | N |
| *Craugastor angelicus* | Neotropical | Costa Rica | 947 | 348.1 | 18.2 | 6.69 | N |
| *Craugastor catalinae* | Neotropical | Costa Rica, Panama | 1284 | 411.9 | 14.2 | 4.49 | N |
| *Craugastor coffeus* | Neotropical | Guatemala | 97 | 0.0 |  | 0.00 | N |
| *Craugastor cruzi* | Neotropical | Honduras | 7 | 6.6 | 25.0 | 22.60 | N |
| *Craugastor emcelae* | Neotropical | Costa Rica, Panama | 1767 | 1667.3 | 13.1 | 12.36 | N |
| *Craugastor emleni* | Neotropical | Honduras | 799 | 96.5 | 25.0 | 3.02 | N |
| *Craugastor epochthidius* | Neotropical | Honduras | 457 | 448.8 | 25.0 | 24.53 | N |
| *Craugastor fecundus* | Neotropical | Honduras | 142 | 136.2 | 25.0 | 23.94 | N |
| *Craugastor fleischmanni* | Neotropical | Costa Rica | 3285 | 1082.5 | 18.2 | 6.00 | N |
| *Craugastor glaucus* | Neotropical | Mexico | 53 | 0.0 | 5.9 | 0.00 | Y |
| *Craugastor greggi* | Neotropical | Guatemala, Mexico | 59 | 6.9 | 5.9 | 0.70 | N |
| *Craugastor guerreroensis* | Neotropical | Mexico | 7 | 0.0 | 5.9 | 0.00 | N |
| *Craugastor lineatus* | Neotropical | Guatemala, Mexico | 15887 | 659.4 | 22.6 | 0.95 | N |
| *Craugastor megalotympanum* | Neotropical | Mexico | 10 | 10.5 | 5.9 | 5.90 | Y |
| *Craugastor merendonensis* | Neotropical | Honduras | 43 | 0.0 | 25.0 | 0.00 | N |
| *Craugastor milesi* | Neotropical | Honduras | 417 | 112.3 | 25.0 | 6.73 | N |
| *Craugastor olanchano* | Neotropical | Honduras | 596 | 0.0 | 25.0 | 0.00 | N |
| *Craugastor omoaensis* | Neotropical | Honduras | 77 | 0.0 | 25.0 | 0.00 | N |
| *Craugastor polymniae* | Neotropical | Mexico | 303 | 0.0 | 5.9 | 0.00 | N |
| *Craugastor pozo* | Neotropical | Mexico | 13 | 3.9 | 5.9 | 1.82 | Y |
| *Craugastor ranoides* | Neotropical | Costa Rica, Panama, Nicaragua | 40670 | 13336.7 | 13.4 | 4.26 | N |
| *Craugastor saltuarius* | Neotropical | Honduras | 294 | 224.6 | 25.0 | 19.12 | N |
| *Craugastor stadelmani* | Neotropical | Honduras | 793 | 324.3 | 25.0 | 10.23 | N |
| *Craugastor tabasarae* | Neotropical | Panama | 28 | 18.9 | 13.1 | 8.77 | N |
| *Craugastor taurus* | Neotropical | Costa Rica, Panama | 5852 | 1737.0 | 18.2 | 5.34 | N |
| *Craugastor trachydermus* | Neotropical | Guatemala | 51 | 7.6 | 30.8 | 4.62 | N |
| *Cryptobatrachus nicefori* | Neotropical | Colombia | 76 | 75.8 | 7.6 | 7.60 | N |
| *Cycloramphus faustoi* | Neotropical | Brazil | 12 | 12.3 | 7.8 | 7.80 | N |
| *Dendropsophus amicorum* | Neotropical | Venezuela | 23 | 11.9 | 12.4 | 6.42 | N |
| *Duellmanohyla salvavida* | Neotropical | Honduras | 327 | 245.0 | 25.0 | 18.72 | N |
| *Duellmanohyla soralia* | Neotropical | Guatemala, Honduras | 1031 | 391.8 | 28.1 | 10.64 | N |
| *Duellmanohyla uranochroa* | Neotropical | Costa Rica, Panama | 14599 | 7704.6 | 16.9 | 8.97 | N |
| *Duttaphrynus sumatranus* | Indomalayan | Indonesia | 88 | 0.0 | N/A | N/A | N |
| *Ecnomiohyla echinata* | Neotropical | Mexico | 32 | 0.0 | 5.9 | 0.00 | N |
| *Ecnomiohyla rabborum* | Neotropical | Panama | 4 | 0.0 | 13.1 | 0.00 | N |
| *Ecnomiohyla salvaje* | Neotropical | Guatemala, Honduras | 52 | 0.0 |  | 0.00 | N |
| *Ecnomiohyla valancifer* | Neotropical | Mexico | 1248 | 999.6 | 5.9 | 4.72 | N |
| *Eleutherodactylus albipes* | Neotropical | Cuba | 35 | 22.6 | 5.0 | 3.20 | Y |
| *Eleutherodactylus alticola* | Neotropical | Jamaica | 53 | 51.9 | N/A | N/A | Y |
| *Eleutherodactylus amadeus* | Neotropical | Haiti | 368 | 0.0 | 0.0 | 0.00 | N |
| *Eleutherodactylus apostates* | Neotropical | Haiti | 455 | 0.0 | 0.0 | 0.00 | N |
| *Eleutherodactylus bakeri* | Neotropical | Haiti | 455 | 0.0 | 0.0 | 0.00 | N |
| *Eleutherodactylus bartonsmithi* | Neotropical | Cuba | 44 | 3.1 | 5.0 | 0.35 | Y |
| *Eleutherodactylus blairhedgesi* | Neotropical | Cuba | 10 | 3.3 | 5.0 | 1.69 | Y |
| *Eleutherodactylus bresslerae* | Neotropical | Cuba | 15 | 8.7 | 5.0 | 2.90 | N |
| *Eleutherodactylus brevirostris* | Neotropical | Haiti | 475 | 0.0 | 0.0 | 0.00 | N |
| *Eleutherodactylus caribe* | Neotropical | Haiti | 15 | 0.0 | 0.0 | 0.00 | N |
| *Eleutherodactylus cavernicola* | Neotropical | Jamaica | 84 | 83.6 | N/A | N/A | Y |
| *Eleutherodactylus chlorophenax* | Neotropical | Haiti | 455 | 0.0 | 0.0 | 0.00 | N |
| *Eleutherodactylus corona* | Neotropical | Haiti | 11 | 0.0 | 0.0 | 0.00 | N |
| *Eleutherodactylus cubanus* | Neotropical | Cuba | 126 | 112.2 | 5.0 | 4.44 | Y |
| *Eleutherodactylus darlingtoni* | Neotropical | Haiti | 74 | 0.0 | 0.0 | 0.00 | N |
| *Eleutherodactylus dixoni* | Neotropical | Mexico | 142 | 0.0 | 5.9 | 0.00 | N |
| *Eleutherodactylus dolomedes* | Neotropical | Haiti | 11 | 0.0 | 0.0 | 0.00 | N |
| *Eleutherodactylus eneidae* | Neotropical | Puerto Rico | 1769 | 183.0 | N/A | N/A | N |
| *Eleutherodactylus eunaster* | Neotropical | Haiti | 455 | 0.0 | 0.0 | 0.00 | N |
| *Eleutherodactylus fowleri* | Neotropical | Dominican Republic, Haiti | 109 | 53.9 | 15.8 | 7.81 | N |
| *Eleutherodactylus furcyensis* | Neotropical | Dominican Republic, Haiti | 1765 | 139.3 | 15.8 | 1.25 | N |
| *Eleutherodactylus fuscus* | Neotropical | Jamaica | 380 | 58.6 | N/A | N/A | Y |
| *Eleutherodactylus glandulifer* | Neotropical | Haiti | 673 | 0.0 | 0.0 | 0.00 | N |
| *Eleutherodactylus glanduliferoides* | Neotropical | Haiti | 38 | 0.0 | 0.0 | 0.00 | N |
| *Eleutherodactylus grandis* | Neotropical | Mexico | 46 | 0.0 | 5.9 | 0.00 | Y |
| *Eleutherodactylus griphus* | Neotropical | Jamaica | 165 | 101.1 | N/A | N/A | N |
| *Eleutherodactylus iberia* | Neotropical | Cuba | 85 | 84.7 | 5.0 | 5.00 | N |
| *Eleutherodactylus jasperi* | Neotropical | Puerto Rico | 144 | 26.9 | N/A | N/A | N |
| *Eleutherodactylus jaumei* | Neotropical | Cuba | 38 | 28.4 | 5.0 | 3.74 | N |
| *Eleutherodactylus juanariveroi* | Neotropical | Puerto Rico | 5 | 0.0 | N/A | N/A | Y |
| *Eleutherodactylus jugans* | Neotropical | Dominican Republic, Haiti | 1189 | 183.8 | 15.8 | 2.44 | N |
| *Eleutherodactylus junori* | Neotropical | Jamaica | 361 | 36.8 | N/A | N/A | N |
| *Eleutherodactylus karlschmidti* | Neotropical | Puerto Rico | 1119 | 72.0 | N/A | N/A | N |
| *Eleutherodactylus lamprotes* | Neotropical | Haiti | 455 | 0.0 | 0.0 | 0.00 | N |
| *Eleutherodactylus leoncei* | Neotropical | Dominican Republic, Haiti | 1807 | 426.3 | 15.8 | 3.73 | N |
| *Eleutherodactylus locustus* | Neotropical | Puerto Rico | 509 | 92.9 | N/A | N/A | Y |
| *Eleutherodactylus lucioi* | Neotropical | Haiti | 35 | 0.0 | 0.0 | 0.00 | N |
| *Eleutherodactylus mariposa* | Neotropical | Cuba | 142 | 62.3 | 5.0 | 2.19 | N |
| *Eleutherodactylus nortoni* | Neotropical | Dominican Republic, Haiti | 2583 | 107.1 | 15.8 | 0.66 | N |
| *Eleutherodactylus orcutti* | Neotropical | Jamaica | 285 | 151.6 | N/A | N/A | N |
| *Eleutherodactylus orientalis* | Neotropical | Cuba | 18 | 18.3 | 5.0 | 5.00 | Y |
| *Eleutherodactylus oxyrhyncus* | Neotropical | Dominican Republic, Haiti | 1415 | 0.0 | 15.8 | 0.00 | N |
| *Eleutherodactylus parabates* | Neotropical | Dominican Republic, Haiti | 220 | 0.0 | 15.8 | 0.00 | N |
| *Eleutherodactylus parapelates* | Neotropical | Haiti | 606 | 0.0 | 0.0 | 0.00 | N |
| *Eleutherodactylus paulsoni* | Neotropical | Haiti | 3767 | 0.0 | 0.0 | 0.00 | N |
| *Eleutherodactylus pezopetrus* | Neotropical | Cuba | 22 | 0.0 | 5.0 | 0.00 | N |
| *Eleutherodactylus poolei* | Neotropical | Haiti | 19 | 0.0 | 0.0 | 0.00 | Y |
| *Eleutherodactylus rhodesi* | Neotropical | Haiti | 85 | 0.0 | 0.0 | 0.00 | N |
| *Eleutherodactylus richmondi* | Neotropical | Puerto Rico | 292 | 59.5 | N/A | N/A | N |
| *Eleutherodactylus rivularis* | Neotropical | Cuba | 86 | 4.3 | 5.0 | 0.25 | Y |
| *Eleutherodactylus rufescens* | Neotropical | Mexico | 5 | 0.0 | 5.9 | 0.00 | N |
| *Eleutherodactylus rufifemoralis* | Neotropical | Dominican Republic | 894 | 23.4 | 15.8 | 0.41 | N |
| *Eleutherodactylus schmidti* | Neotropical | Dominican Republic, Haiti | 12089 | 282.6 | 15.8 | 0.37 | Y |
| *Eleutherodactylus sciagraphus* | Neotropical | Haiti | 77 | 0.0 | 0.0 | 0.00 | N |
| *Eleutherodactylus semipalmatus* | Neotropical | Haiti | 1087 | 0.0 | 0.0 | 0.00 | N |
| *Eleutherodactylus sisyphodemus* | Neotropical | Jamaica | 36 | 31.2 | N/A | N/A | N |
| *Eleutherodactylus symingtoni* | Neotropical | Cuba | 236 | 67.7 | 5.0 | 1.43 | Y |
| *Eleutherodactylus tetajulia* | Neotropical | Cuba | 71 | 71.3 | 5.0 | 5.00 | N |
| *Eleutherodactylus thorectes* | Neotropical | Haiti | 41 | 0.0 | 0.0 | 0.00 | N |
| *Eleutherodactylus tonyi* | Neotropical | Cuba | 19 | 5.6 | 5.0 | 1.45 | Y |
| *Eleutherodactylus turquinensis* | Neotropical | Cuba | 270 | 216.0 | 5.0 | 4.00 | Y |
| *Eleutherodactylus ventrilineatus* | Neotropical | Haiti | 41 | 0.0 | 0.0 | 0.00 | N |
| *Eleutherodactylus warreni* | Neotropical | Haiti | 180 | 0.0 | 0.0 | 0.00 | N |
| *Eupsophus insularis* | Neotropical | Chile | 53 | 24.2 | 37.9 | 17.29 | N |
| *Exerodonta perkinsi* | Neotropical | Guatemala | 34 | 0.0 | 30.8 | 0.00 | N |
| *Fejervarya murthii* | Indomalayan | India | 31 | 0.2 | 8.0 | 0.06 | N |
| *Gastrotheca lauzuricae* | Neotropical | Bolivia | 194 | 166.6 | 8.1 | 6.95 | N |
| *Gastrotheca zeugocystis* | Neotropical | Peru | 29 | 0.0 | 15.5 | 0.00 | N |
| *Geocrinia alba* | Australasian | Australia | 196 | 48.3 | 9.4 | 2.32 | Y |
| *Glandirana minima* | Palearctic | China | 3928 | 0.0 | N/A | N/A | Y |
| *Heleophryne rosei* | Afrotropical | South Africa | 18 | 15.7 | 47.2 | 40.29 | Y |
| *Holoaden bradei* | Neotropical | Brazil | 363 | 323.4 | 7.8 | 6.96 | Y |
| *Hyalinobatrachium crybetes* | Neotropical | Honduras | 108 | 60.5 | 25.0 | 13.96 | N |
| *Hyla bocourti* | Neotropical | Guatemala | 714 | 22.6 | 30.8 | 0.97 | N |
| *Hyla heinzsteinitzi* | Palearctic | Israel, Palestine | 45 | 0.3 | N/A | N/A | N |
| *Hylomantis lemur* | Neotropical | Colombia, Costa Rica Panama | N/A | N/A | N/A | N/A | N |
| *Hyloscirtus chlorosteus* | Neotropical | Bolivia | 148 | 58.2 | 8.1 | 3.19 | N |
| *Hyloscirtus colymba* | Neotropical | Costa Rica, Panama | 16545 | 11708.5 | 13.6 | 9.91 | N |
| *Hyloscirtus ptychodactylus* | Neotropical | Ecuador | 488 | 341.5 | 27.6 | 19.30 | N |
| *Hyloxalus anthracinus* | Neotropical | Ecuador | 2262 | 1.2 | 27.6 | 0.01 | N |
| *Hyloxalus delatorreae* | Neotropical | Ecuador | 184 | 14.9 | 27.6 | 2.24 | N |
| *Hyloxalus edwardsi* | Neotropical | Colombia | 49 | 0.0 | 7.6 | 0.00 | N |
| *Hyloxalus ruizi* | Neotropical | Colombia | 81 | 0.0 | 7.6 | 0.00 | N |
| *Hyloxalus vertebralis* | Neotropical | Ecuador | 5981 | 290.5 | 27.6 | 1.34 | N |
| *Hyperolius pickersgilli* | Afrotropical | South Africa | 2313 | 164.1 | 47.2 | 3.35 | Y |
| *Hyperolius watsonae* | Afrotropical | Tanzania | 7 | 4.4 | 36.7 | 21.75 | N |
| *Hypodactylus lucida* | Neotropical | Peru | 66 | 0.0 | 15.5 | 0.00 | N |
| *Hypsiboas cymbalum* | Neotropical | Brazil | 210 | 0.0 | 7.8 | 0.00 | N |
| *Incilius cristatus* | Neotropical | Mexico | 101 | 0.0 | 5.9 | 0.00 | Y |
| *Incilius fastidiosus* | Neotropical | Costa Rica, Panama | 323 | 304.3 | 16.7 | 16.04 | N |
| *Incilius peripatetes* | Neotropical | Panama | 129 | 24.3 | 13.1 | 2.47 | N |
| *Indirana gundia* | Indomalayan | India | 69 | 0.0 | 8.0 | 0.00 | Y |
| *Indirana phrynoderma* | Indomalayan | India | 93 | 92.9 | 8.0 | 8.00 | N |
| *Ingerana charlesdarwini* | Indomalayan | India | 59 | 0.0 | 8.0 | 0.00 | N |
| *Insuetophrynus acarpicus* | Neotropical | Chile | 122 | 21.4 | 37.9 | 6.65 | N |
| *Isthmohyla angustilineata* | Neotropical | Costa Rica, Panama | 1496 | 485.9 | 18.1 | 5.84 | N |
| *Isthmohyla calypsa* | Neotropical | Costa Rica, Panama | 321 | 233.4 | 13.7 | 10.18 | N |
| *Isthmohyla debilis* | Neotropical | Costa Rica, Panama | 1995 | 1215.3 | 13.7 | 8.53 | N |
| *Isthmohyla graceae* | Neotropical | Costa Rica, Panama | 1887 | 586.9 |  | 0.00 | N |
| *Isthmohyla insolita* | Neotropical | Honduras | 92 | 64.8 | 25.0 | 17.63 | N |
| *Isthmohyla rivularis* | Neotropical | Costa Rica, Panama | 6984 | 2847.5 | 17.1 | 6.93 | N |
| *Isthmohyla tica* | Neotropical | Costa Rica, Panama | 6005 | 2659.0 | 17.4 | 7.70 | N |
| *Leiopelma archeyi* | Australasian | New Zealand | 759 | 404.4 | 7.9 | 4.21 | N |
| *Leptobrachella palmata* | Indomalayan | Malaysia | 99 | 33.9 | N/A | N/A | N |
| *Leptodactylodon erythrogaster* | Afrotropical | Cameroon | 33 | 8.1 | N/A | N/A | N |
| *Leptodactylus fallax* | Neotropical | Dominican Republic, Montserrat | 214 | 14.8 | 15.8 | 1.09 | N |
| *Leptodactylus magistris* | Neotropical | Venezuela | 36 | 22.6 | 12.4 | 7.89 | N |
| *Leptodactylus silvanimbus* | Neotropical | Honduras | 138 | 77.6 | 25.0 | 14.08 | N |
| *Leptophryne cruentata* | Indomalayan | Indonesia | 707 | 153.6 | N/A | N/A | Y |
| *Lithobates chichicuahutla* | Neotropical | Mexico | 4 | 0.0 | 5.9 | 0.00 | N |
| *Lithobates omiltemanus* | Neotropical | Mexico | 1090 | 0.0 | 5.9 | 0.00 | N |
| *Lithobates pueblae* | Neotropical | Mexico | 8 | 0.0 | 5.9 | 0.00 | Y |
| *Lithobates sevosus* | Nearctic | United States | 21459 | 1153.6 | 7.4 | 0.40 | N |
| *Lithobates subaquavocalis* | Nearctic | United States | 223 | 71.2 | 7.4 | 2.37 | N |
| *Lithobates tlaloci* | Neotropical | Mexico | 37 | 0.0 | 5.9 | 0.00 | Y |
| *Lithobates vibicarius* | Neotropical | Costa Rica, Panama | 2713 | 1382.0 | 17.1 | 8.66 | N |
| *Litoria booroolongensis* | Australasian | Australia | 136078 | 21073.0 | 9.4 | 1.46 | N |
| *Litoria castanea* | Australasian | Australia | 8536 | 763.6 | 9.4 | 0.84 | N |
| *Litoria lorica* | Australasian | Australia | 1186 | 435.4 | 9.4 | 3.45 | Y |
| *Litoria myola* | Australasian | Australia | 4 | 0.3 | 9.4 | 0.63 | N |
| *Litoria nyakalensis* | Australasian | Australia | 11680 | 5215.0 | 9.4 | 4.20 | Y |
| *Litoria piperata* | Australasian | Australia | 5039 | 1087.7 | 9.4 | 2.03 | N |
| *Litoria spenceri* | Australasian | Australia | 16621 | 7616.8 | 9.4 | 4.31 | Y |
| *Mannophryne caquetio* | Neotropical | Venezuela | 84 | 70.0 | 12.4 | 10.36 | Y |
| *Mannophryne cordilleriana* | Neotropical | Venezuela | 92 | 43.4 | 12.4 | 5.84 | N |
| *Mannophryne lamarcai* | Neotropical | Venezuela | 50 | 30.6 | 12.4 | 7.61 | N |
| *Mannophryne neblina* | Neotropical | Venezuela | 96 | 80.1 | 12.4 | 10.35 | N |
| *Mannophryne olmonae* | Neotropical | Trinidad, Tobago | 101 | 56.1 | N/A | N/A | N |
| *Mantella aurantiaca* | Afrotropical | Madagascar | 541 | 13.8 | 5.0 | 0.13 | N |
| *Mantella cowanii* | Afrotropical | Madagascar | 254 | 9.7 | 5.0 | 0.19 | N |
| *Mantella milotympanum* | Afrotropical | Madagascar | 51 | 15.2 | 5.0 | 1.50 | N |
| *Mantidactylus pauliani* | Afrotropical | Madagascar | 257 | 0.0 | 5.0 | 0.00 | N |
| *Megastomatohyla mixe* | Neotropical | Mexico | 33 | 0.0 | 5.9 | 0.00 | N |
| *Megastomatohyla pellita* | Neotropical | Mexico | 1093 | 0.0 | 5.9 | 0.00 | N |
| *Melanophryniscus langonei* | Neotropical | Uruguay | 205 | 0.0 | 8.1 | 0.00 | N |
| *Micrixalus kottigeharensis* | Indomalayan | India | 399 | 0.0 | 8.0 | 0.00 | N |
| *Microbatrachella capensis* | Afrotropical | South Africa | 1363 | 348.2 | 47.2 | 12.06 | Y |
| *Microhyla karunaratnei* | Indomalayan | Sri Lanka | 43 | 3.6 | N/A | N/A | N |
| *Minyobates steyermarki* | Neotropical | Venezuela | 24 | 23.7 | 12.4 | 12.40 | N |
| *Nannophrys marmorata* | Indomalayan | Sri Lanka | 81 | 0.0 | N/A | N/A | Y |
| *Nectophrynoides paulae* | Afrotropical | Tanzania | 18 | 17.2 | 36.7 | 34.96 | N |
| *Nectophrynoides poyntoni* | Afrotropical | Tanzania | 3 | 3.1 | 36.7 | 36.70 | N |
| *Nectophrynoides wendyae* | Afrotropical | Tanzania | 15 | 15.1 | 36.7 | 36.70 | N |
| *Niceforonia adenobrachia* | Neotropical | Colombia | 97 | 47.9 | 7.6 | 3.76 | N |
| *Nimbaphrynoides liberiensis* | Palearctic | Liberia | 62 | 62.0 | N/A | N/A | N |
| *Nimbaphrynoides occidentalis* | Afrotropical | Côte d'Ivoire, Guinea | 111 | 102.3 | N/A | N/A | N |
| *Nymphargus anomalus* | Neotropical | Ecuador | 12 | 0.3 | 27.6 | 0.59 | N |
| *Nymphargus laurae* | Neotropical | Ecuador | N/A | N/A | 27.6 | N/A | N |
| *Odontophrynus moratoi* | Neotropical | Brazil | 111 | 0.0 | 7.8 | 0.00 | N |
| *Odorrana wuchuanensis* | Palearctic | China | 525 | 0.0 | N/A | N/A | Y |
| *Oophaga lehmanni* | Neotropical | Colombia | 243 | 187.6 | 7.6 | 5.88 | N |
| *Oreobates pereger* | Neotropical | Peru | 37 | 0.0 | 15.5 | 0.00 | N |
| *Oreobates zongoensis* | Neotropical | Bolivia | 16 | 0.0 | 8.1 | 0.00 | N |
| *Oreolalax liangbeiensis* | Palearctic | China | 78 | 48.2 | N/A | N/A | N |
| *Parhoplophryne usambarica* | Afrotropical | Tanzania | 11 | 3.3 | 36.7 | 11.60 | N |
| *Pelophryne linanitensis* | Indomalayan | Indonesia, Malaysia | 300 | 82.9 | N/A | N/A | N |
| *Pelophryne murudensis* | Indomalayan | Malaysia | 322 | 88.5 | N/A | N/A | N |
| *Pelophylax cerigensis* | Palearctic | Greece | 4 | 0.0 | N/A | N/A | N |
| *Peltophryne florentinoi* | Neotropical | Cuba | 69 | 68.7 | 5.0 | 5.00 | N |
| *Peltophryne fluviatica* | Neotropical | Dominican Republic | 435 | 0.0 | 15.8 | 0.00 | N |
| *Peltophryne lemur* | Neotropical | British Virgin Islands, Puerto Rico | 1235 | 76.5 | 1.0 | 0.06 | Y |
| *Petropedetes dutoiti* | Afrotropical | Kenya, Uganda | 774 | 512.8 | 66.1 | 43.79 | N |
| *Philautus jacobsoni* | Indomalayan | Indonesia | 212 | 33.3 | N/A | N/A | N |
| *Philautus sanctisilvaticus* | Indomalayan | India | 84 | 0.0 | 8.0 | 0.00 | Y |
| *Philoria frosti* | Australasian | Australia | 103 | 71.4 | 9.4 | 6.49 | Y |
| *Phrynobatrachus intermedius* | Afrotropical | Ghana | N/A | N/A | N/A | N/A | N |
| *Phrynopus dagmarae* | Neotropical | Peru | 669 | 0.0 | 15.5 | 0.00 | N |
| *Phrynopus heimorum* | Neotropical | Peru | 39 | 0.0 | 15.5 | 0.00 | N |
| *Phrynopus juninensis* | Neotropical | Peru | 80 | 0.0 | 15.5 | 0.00 | N |
| *Phrynopus kauneorum* | Neotropical | Peru | 472 | 0.0 | 15.5 | 0.00 | N |
| *Phrynopus tautzorum* | Neotropical | Peru | 155 | 0.0 | 15.5 | 0.00 | N |
| *Phyllodytes auratus* | Neotropical | Trinidad, Tobago | 18 | 6.3 | N/A | N/A | N |
| *Phyllomedusa ayeaye* | Neotropical | Brazil | 91 | 0.0 | 7.8 | 0.00 | N |
| *Platymantis insulatus* | Indomalayan | Philippines | 6 | 0.0 | 53.2 | 0.00 | N |
| *Plectrohyla acanthodes* | Neotropical | Guatemala, Mexico | 995 | 306.4 | 30.8 | 12.32 | N |
| *Plectrohyla avia* | Neotropical | Guatemala, Mexico | 2618 | 603.9 | 16.9 | 3.92 | N |
| *Plectrohyla calthula* | Neotropical | Mexico | 17 | 0.0 | 5.9 | 0.00 | N |
| *Plectrohyla calvicollina* | Neotropical | Mexico | 52 | 0.0 | 5.9 | 0.00 | N |
| *Plectrohyla celata* | Neotropical | Mexico | 204 | 0.0 | 5.9 | 0.00 | N |
| *Plectrohyla cembra* | Neotropical | Mexico | 255 | 0.0 | 5.9 | 0.00 | N |
| *Plectrohyla chryses* | Neotropical | Mexico | 234 | 0.0 | 5.9 | 0.00 | N |
| *Plectrohyla chrysopleura* | Neotropical | Honduras | 111 | 100.1 | 25.0 | 22.56 | N |
| *Plectrohyla crassa* | Neotropical | Mexico | 79 | 0.0 | 5.9 | 0.00 | N |
| *Plectrohyla cyanomma* | Neotropical | Mexico | 62 | 0.0 | 5.9 | 0.00 | N |
| *Plectrohyla dasypus* | Neotropical | Honduras | 262 | 61.1 | 25.0 | 5.84 | N |
| *Plectrohyla ephemera* | Neotropical | Mexico | 3 | 0.0 | 5.9 | 0.00 | N |
| *Plectrohyla exquisita* | Neotropical | Honduras | 133 | 66.8 | 25.0 | 12.59 | N |
| *Plectrohyla guatemalensis* | Neotropical | El Salvador, Guatemala, Honduras, Mexico | 55405 | 5179.0 | 26.6 | 2.52 | N |
| *Plectrohyla hartwegi* | Neotropical | Guatemala, Mexico | 5508 | 2015.4 | 27.6 | 10.10 | N |
| *Plectrohyla hazelae* | Neotropical | Mexico | 501 | 0.0 | 5.9 | 0.00 | N |
| *Plectrohyla ixil* | Neotropical | Guatemala | 416 | 146.3 | 30.8 | 10.90 | N |
| *Plectrohyla pachyderma* | Neotropical | Mexico | 15 | 0.0 | 5.9 | 0.00 | Y |
| *Plectrohyla pokomchi* | Neotropical | Guatemala | 1221 | 536.6 | 30.8 | 13.54 | N |
| *Plectrohyla psarosema* | Neotropical | Mexico | 11 | 0.0 | 5.9 | 0.00 | N |
| *Plectrohyla pycnochila* | Neotropical | Mexico | 30 | 0.0 | 5.9 | 0.00 | N |
| *Plectrohyla quecchi* | Neotropical | Guatemala | 7110 | 1386.5 | 30.8 | 6.01 | N |
| *Plectrohyla sabrina* | Neotropical | Mexico | 26 | 0.0 | 5.9 | 0.00 | Y |
| *Plectrohyla siopela* | Neotropical | Mexico | 16 | 16.2 | 5.9 | 5.90 | N |
| *Plectrohyla tecunumani* | Neotropical | Guatemala | 24 | 17.0 | 30.8 | 21.75 | N |
| *Plectrohyla teuchestes* | Neotropical | Guatemala | 15 | 0.0 | 30.8 | 0.00 | N |
| *Plectrohyla thorectes* | Neotropical | Mexico | 342 | 0.0 | 5.9 | 0.00 | N |
| *Polypedates fastigo* | Indomalayan | Sri Lanka | 3 | 0.0 | N/A | N/A | N |
| *Pristimantis albericoi* | Neotropical | Colombia | 3 | 0.0 | 7.6 | 0.00 | N |
| *Pristimantis bernali* | Neotropical | Colombia | 227 | 0.0 | 7.6 | 0.00 | N |
| *Pristimantis hamiotae* | Neotropical | Ecuador | 2 | 0.0 | 27.6 | 0.00 | N |
| *Pristimantis lichenoides* | Neotropical | Colombia | 63 | 0.0 | 7.6 | 0.00 | N |
| *Pristimantis phragmipleuron* | Neotropical | Colombia | 160 | 0.0 | 7.6 | 0.00 | N |
| *Pristimantis simonsii* | Neotropical | Peru | 78 | 0.0 | 15.5 | 0.00 | N |
| *Pristimantis torrenticola* | Neotropical | Colombia | 4 | 0.0 | 7.6 | 0.00 | N |
| *Pristimantis tribulosus* | Neotropical | Colombia | 4 | 0.0 | 7.6 | 0.00 | N |
| *Pristimantis veletis* | Neotropical | Colombia | 213 | 0.0 | 7.6 | 0.00 | N |
| *Prostherapis dunni* | Neotropical | Venezuela | 308 | 298.1 | 12.4 | 11.98 | Y |
| *Pseudophilautus amboli* | Indomalayan | India | 91 | 0.0 | 8.0 | 0.00 | Y |
| *Pseudophilautus limbus* | Indomalayan | Sri Lanka | 8 | 8.2 | N/A | N/A | N |
| *Pseudophilautus lunatus* | Indomalayan | Sri Lanka | 2 | 0.0 | N/A | N/A | N |
| *Pseudophilautus macropus* | Indomalayan | Sri Lanka | 13 | 0.0 | N/A | N/A | N |
| *Pseudophilautus nemus* | Indomalayan | Sri Lanka | 8 | 8.2 | N/A | N/A | N |
| *Pseudophilautus papillosus* | Indomalayan | Sri Lanka | 2 | 0.0 | N/A | N/A | N |
| *Pseudophilautus procax* | Indomalayan | Sri Lanka | 8 | 0.0 | N/A | N/A | N |
| *Pseudophilautus simba* | Indomalayan | Sri Lanka | 2 | 0.0 | N/A | N/A | N |
| *Pseudophryne corroboree* | Australasian | Australia | 1082 | 1047.3 | 9.4 | 9.10 | Y |
| *Psychrophrynella guillei* | Neotropical | Bolivia | 12 | 12.5 | 8.1 | 8.10 | N |
| *Psychrophrynella illimani* | Neotropical | Bolivia | 8 | 0.0 | 8.1 | 0.00 | N |
| *Psychrophrynella kallawaya* | Neotropical | Bolivia | 12 | 12.5 | 8.1 | 8.10 | N |
| *Psychrophrynella saltator* | Neotropical | Bolivia | 12 | 12.3 | 8.1 | 8.10 | N |
| *Ptychohyla dendrophasma* | Neotropical | Guatemala | 45 | 0.0 | 30.8 | 0.00 | N |
| *Ptychohyla hypomykter* | Neotropical | Guatemala, Honduras, Nicaragua | 20680 | 5179.7 | 15.5 | 3.88 | N |
| *Ptychohyla macrotympanum* | Neotropical | Guatemala | 52 | 0.0 | 30.8 | 0.00 | N |
| *Ptychohyla sanctaecrucis* | Neotropical | Guatemala | 74 | 37.2 | 30.8 | 15.43 | N |
| *Rana chevronta* | Palearctic | China | 336 | 11.6 | N/A | N/A | N |
| *Rana holtzi* | Palearctic | Turkey | 86 | 0.0 | N/A | N/A | Y |
| *Ranitomeya abdita* | Neotropical | Ecuador | 7 | 1.4 | 27.6 | 5.26 | N |
| *Ranitomeya dorisswansonae* | Neotropical | Colombia | 33 | 14.0 | 7.6 | 3.25 | N |
| *Raorchestes chalazodes* | Indomalayan | India | 42 | 0.0 | 8.0 | 0.00 | N |
| *Raorchestes chlorosomma* | Indomalayan | India | N/A | N/A | 8.0 | N/A | Y |
| *Raorchestes griet* | Indomalayan | India | 80 | 0.0 | 8.0 | 0.00 | N |
| *Raorchestes kaikatti* | Indomalayan | India | N/A | N/A | 8.0 | N/A | Y |
| *Raorchestes marki* | Indomalayan | India | N/A | N/A | 8.0 | N/A | Y |
| *Raorchestes munnarensis* | Indomalayan | India | 52 | 0.0 | 8.0 | 0.00 | N |
| *Raorchestes ponmudi* | Indomalayan | India | 10 | 0.3 | 8.0 | 0.23 | N |
| *Raorchestes resplendens* | Indomalayan | India | N/A | N/A | 8.0 | N/A | N |
| *Raorchestes shillongensis* | Indomalayan | India | 80 | 0.0 | 8.0 | 0.00 | N |
| *Rhacophorus pseudomalabaricus* | Indomalayan | India | 116 | 116.3 | 8.0 | 8.00 | N |
| *Rhinella amabilis* | Neotropical | Ecuador | 7 | 0.0 | 27.6 | 0.00 | N |
| *Rhinella chavin* | Neotropical | Peru | 329 | 0.0 | 15.5 | 0.00 | N |
| *Rhinella rostrata* | Neotropical | Colombia | 291 | 0.0 | 7.6 | 0.00 | N |
| *Rhinoderma rufum* | Neotropical | Chile | 24030 | 33.2 | 37.9 | 0.05 | N |
| *Scinax alcatraz* | Neotropical | Brazil | 2 | 0.0 | 7.8 | 0.00 | Y |
| *Scinax faivovichi* | Neotropical | Brazil | 1 | 0.0 | 7.8 | 0.00 | N |
| *Scinax peixotoi* | Neotropical | Brazil | 0 | 0.0 | 7.8 | 0.00 | N |
| *Scutiger maculatus* | Palearctic | China | 2295 | 147.6 | N/A | N/A | N |
| *Somuncuria somuncurensis* | Neotropical | Argentina | 242 | 242.3 | 26.5 | 26.50 | N |
| *Strabomantis helonotus* | Neotropical | Ecuador | 199 | 0.0 | 27.6 | 0.00 | N |
| *Stumpffia helenae* | Afrotropical | Madagascar | 293 | 0.0 | 5.0 | 0.00 | N |
| *Taudactylus acutirostris* | Australasian | Australia | 14665 | 6524.5 | 9.4 | 4.18 | Y |
| *Taudactylus eungellensis* | Australasian | Australia | 337 | 158.5 | 9.4 | 4.43 | Y |
| *Taudactylus pleione* | Australasian | Australia | 127 | 86.4 | 9.4 | 6.41 | Y |
| *Taudactylus rheophilus* | Australasian | Australia | 4742 | 1880.5 | 9.4 | 3.73 | Y |
| *Telmatobius atacamensis* | Neotropical | Argentina | 20 | 19.5 | 26.5 | 26.50 | N |
| *Telmatobius cirrhacelis* | Neotropical | Ecuador | 8 | 4.0 | 27.6 | 13.85 | N |
| *Telmatobius culeus* | Neotropical | Bolivia, Peru | 17540 | 10994.3 | 15.5 | 10.03 | Y |
| *Telmatobius espadai* | Neotropical | Bolivia | 14603 | 4607.7 | 8.1 | 2.56 | N |
| *Telmatobius gigas* | Neotropical | Bolvia | 58 | 0.0 | 8.1 | 0.00 | N |
| *Telmatobius niger* | Neotropical | Ecuador | 5994 | 165.3 | 27.6 | 0.76 | N |
| *Telmatobius pefauri* | Neotropical | Chile | 105 | 0.0 | 37.9 | 0.00 | N |
| *Telmatobius punctatus* | Neotropical | Peru | 81 | 0.0 | 15.5 | 0.00 | N |
| *Telmatobius vellardi* | Neotropical | Ecuador | 172 | 0.0 | 27.6 | 0.00 | N |
| *Telmatobius zapahuirensis* | Neotropical | Chile | 105 | 0.0 | 37.9 | 0.00 | N |
| *Telmatobufo bullocki* | Neotropical | Chile | 4678 | 64.9 | 37.9 | 0.53 | N |
| *Vandijkophrynus amatolicus* | Afrotropical | South Africa | 1807 | 69.5 | 47.2 | 1.82 | N |
| *Werneria iboundji* | Afrotropical | Gabon | 10 | 0.0 | N/A | N/A | N |
| *Wolterstorffina chirioi* | Afrotropical | Cameroon | 31 | 21.7 | N/A | N/A | N |
| *Xanthophryne tigerina* | Indomalayan | India | N/A | N/A | 8.0 | N/A | Y |
| *Xenopus itombwensis* | Afrotropical | Congo | 40 | 40.3 | N/A | N/A | N |
| *Xenopus longipes* | Afrotropical | Cameroon | 3 | 0.0 | N/A | N/A | N |
