# Supplementary material for: Tourism and the Conservation of Critically Endangered Frogs
Source: PLoS One. 2012 Sep 12;7(9):e43757. doi: 10.1371/journal.pone.0043757 (PMC3440435; doi:10.1371/journal.pone.0043757)
Supplement: Table S2 — This table presents the financial contributions to protected areas from different funding sources in each country where data are available. (DOC) [file pone.0043757.s002.doc]

**Table S2**. Financial contributions to protected area budgets from different funding sources in each country where data are available.

| **Country** | **Government contribution** | **Tourism revenue** | **Donor / other funding** | **Total budget** | **Proportion from tourism [R]** | **Source^1^** |
| --- | --- | --- | --- | --- | --- | --- |
| Argentina* | 16,610,320 | 8,297,163 | 6,402,101 | 31,309,584 | 26.5 | Bovarnick *et al.* 2010 |
| Australia | 1,174,133,308 | 138,595,744 | 154,408,041 | 1,467,137,094 | 9.4 | ACA reports (2006-2009) |
| Bolivia* | 73,041 | 414,256 | 4,615,356 | 5,102,653 | 8.1 | Bovarnick *et al.* 2010 |
| Brazil* | 104,691,819 | 8,156,681 | 20,566,539 | 133,415,039 | 7.8 | Bovarnick *et al.* 2010 |
| British Virgin Islands* | 585,000 | 863,262 | 34,443 | 1,482,705 | 58.2 | Gardner *et al.* 2008 |
| Chile* | 5,705,515 | 3,488,824 | - | 9,194,339 | 37.9 | Bovarnick *et al.* 2010 |
| Colombia* | 12,600,584 | 1,534,291 | 6,031,386 | 20,166,261 | 7.6 | Bovarnick *et al*. 2010 |
| Costa Rica* | 14,302,092 | 5,398,612 | 9,945,245 | 29,645,949 | 18.2 | Bovarnick *et al*. 2010 |
| Cuba* | 2,259,551 | 843,733 | 13,832,772 | 16,936,056 | 5.0 | Bovarnick *et al*. 2010 |
| Dominican Republic* | 7,103,393 | 1,643,612 | 1,633,066 | 10,380,071 | 15.8 | Bovarnick *et al*. 2010 |
| Ecuador* | 1,160,000 | 1,100,000 | 1,517,600 | 3,777,600 | 27.6 | Bovarnick *et al*. 2010 |
| El Salvador* | 395,404 | 26,454 | 3,381,365 | 3,803,223 | 6.9 | Bovarnick *et al*. 2010 |
| Guatemala* | 4,353,715 | 3,707,295 | 3,985,790 | 12,046,800 | 30.8 | Bovarnick *et al*. 2010 |
| Honduras* | 677,057 | 1,032,265 | 2,413,230 | 4,122,552 | 25.0 | Bovarnick *et al*. 2010 |
| Kenya | 743,480,500 | 2,116,934,500 | 342,829,000 | 3,203,244,000 | 66.1 | KWS annual reports (2007/08) |
| Madagascar | 288,000 | 204,000 | 3,600,000 | 4,092,000 | 5.0 | Mansourian and Dudley 2008 |
| Mexico* | 49,046,698 | 4,740,532 | 26,427,009 | 80,214,239 | 5.9 | Bovarnick *et al*. 2010 |
| New Zealand | 284,763,000 | 11,289,000 | 22,769,000 | 318,058,000 | 7.9 | NZ DOC Annual Report to 30 June 2011 |
| Nicaragua* | 576,337 | 441,838 | 4,323,102 | 5,341,277 | 8.3 | Bovarnick *et al*. 2010 |
| Panama* | 1,132,000 | 1,244,755 | 7,130,193 | 9,506,948 | 13.1 | Bovarnick *et al*. 2010 |
| Peru* | 1,810,016 | 2,023,100 | 9,233,984 | 13,067,100 | 15.5 | Bovarnick *et al*. 2010 |
| Philippines | 45,415,544 | 51,269,283 | - | 96,684,827 | 53.0 | C. Custudio pers. comm. 2011 |
| South Africa | 662,483,828 | 724,398,339 | 146,484,530 | 1,533,366,696 | 47.2 | SANParks annual reports, ECPB annual reports, Ezemvelo KZN Wildlife Annual reports |
| Tanzania | 105,775,369,000 | 61,200,000,000 | - | 166,975,369,000 | 36.7 | TANAPA annual reports |
| United States* | 3,160,000,000 | 251,200,000 | - | 3,411,200,000 | 7.4 | J. A. Pendry pers. comm. 2011 |
| Uruguay* | 606,000 | 66,000 | 144,000 | 816,000 | 8.1 | Bovarnick *et al*. 2010 |
| Venezuela* | 20,628,837 | 2,567,260 | - | 20,628,837 | 12.4 | Bovarnick *et al*. 2010 |
| Zimbabwe* | - | 90,038,148 | - | 90,038,148 | 100.0 | ZTA reports 2000-2010 |

* Data in US$, other countries in local currency

** No recent official figures are available for India. Personal communications with parks agency staff on site indicate that R ~ 10% for individual parks, but NGO reports (Guha and Ghosh 2009) suggest that R < 10%. R = 8% provides a conservative estimate.

^1^ ACA = Australian Conservation Agency, ECPB = Eastern Capes Parks Board, KWS = Kenya Wildlife Service, NZ DOC = New Zealand Department of Conservation, SANParks = South Africa National Parks, TANPA = Tanzania National Parks, ZTA = Zimbabwe Tourism Authority

Bovarnick, A., J. Fernandez-Baca, J. Galindo, and H. Negret. 2010. Financial sustainability of protected areas in Latin America and the Caribbean: investment policy guideline. United Nations Development Program and The Nature Conservancy.

Gardner, Lloyd, Smith Abbott, Joseph and Woodfield-Pascoe, Nancy. 2008. British Virgin Islands Protected Areas System Plan 2007-2017. BVI National Parks Trust. Tortola.

Guha, I. and Gosh, S. 2009. A Glimpse of the Tiger: How much are Indians willing to pay for It? SANDEE Working Paper No. 39-09.

Mansourian , S. and N. Dudley. 2008. Public funds to protected areas. Protected Areas for a Living Planet Series. WWF International.
