# Supplementary material for: Tourism and the Conservation of Critically Endangered Frogs
Source: PLoS One. 2012 Sep 12;7(9):e43757. doi: 10.1371/journal.pone.0043757 (PMC3440435; doi:10.1371/journal.pone.0043757)
Supplement: Table S3 — This table presents the CR frog species threatened by tourism activities. (DOC) [file pone.0043757.s003.doc]

**Table S3.** CR frog species threatened by tourism (species from IUCN 2011 Red List).

| **Region** | **Scientific Name** | **Countries** | **Proportion protected by tourism (%)^a^** | **Threat posed by tourism^b^** |
| --- | --- | --- | --- | --- |
| Afrotropical | *Hyperolius pickersgilli* | South Africa | 3.4 | Infrastructure^1^ |
| Afrotropical | *Microbatrachella capensis* | South Africa | 12.1 | Infrastructure^1^ |
| Afrotropical | *Heleophryne rosei* | South Africa | 40.3 | Recreational activities^2^ |
| Australasian | *Geocrinia alba* | Australia | 2.3 | Infrastructure and recreation^1^ |
| Australasian | *Litoria lorica* | Australia | 3.5 | Recreational activities^1^ |
| Australasian | *Taudactylus rheophilus* | Australia | 3.7 | Infrastructure and recreation^1^ |
| Australasian | *Taudactylus acutirostris* | Australia | 4.2 | Infrastructure and recreation^1^ |
| Australasian | *Litoria nyakalensis* | Australia | 4.2 | Recreational activities^1^ |
| Australasian | *Litoria spenceri* | Australia | 4.3 | Recreational activities^1^ |
| Australasian | *Taudactylus eungellensis* | Australia | 4.4 | Infrastructure and recreation^1^ |
| Australasian | *Cophixalus concinnus* | Australia | 6.1 | Infrastructure and recreation^3^ |
| Australasian | *Taudactylus pleione* | Australia | 6.4 | Infrastructure^1^ |
| Australasian | *Philoria frosti* | Australia | 6.5 | Infrastructure and recreation ^1^ |
| Australasian | *Pseudophryne corroboree* | Australia | 9.1 | Infrastructure^1^ |
| Indomalayan | *Indirana gundia* | India | 0.0 | Infrastructure^2^ |
| Indomalayan | *Philautus sanctisilvaticus* | India | 0.0 | Infrastructure^2^ |
| Indomalayan | *Pseudophilautus amboli* | India | 0.0 | Infrastructure^3^ |
| Indomalayan | *Nannophrys marmorata* | Sri Lanka | 0.0 | Recreational activities^3^ |
| Indomalayan | *Raorchestes kaikatti* | India | 0.0 | Infrastructure^2^ |
| Indomalayan | *Raorchestes marki* | India | 0.0 | Infrastructure^2^ |
| Indomalayan | *Xanthophryne tigerina* | India | 0.0 | Infrastructure^3^ |
| Indomalayan | *Raorchestes chlorosomma* | India | 0.0 | Infrastructure and recreation^1^ |
| Indomalayan | *Adenomus dasi* | Sri Lanka | ? | Recreational activities^1^ |
| Indomalayan | *Leptophryne cruentata* | Indonesia | ? | Recreational activities^1^ |
| Neotropical | *Incilius cristatus* | Mexico | 0.0 | Infrastructure^1^ |
| Neotropical | *Alsodes tumultuosus* | Chile | 0.0 | Infrastructure and recreation^3^ |
| Neotropical | *Lithobates pueblae* | Mexico | 0.0 | Infrastructure and recreation^1^ |
| Neotropical | *Lithobates tlaloci* | Mexico | 0.0 | Infrastructure and recreation^1^ |
| Neotropical | *Peltophryne lemur* | British Virgin Islands,  Puerto Rico | 0.0 | Infrastructure and recreation^2^ |
| Neotropical | *Plectrohyla pachyderma* | Mexico | 0.0 | Infrastructure and recreation^2^ |
| Neotropical | *Plectrohyla sabrina* | Mexico | 0.0 | Infrastructure and recreation^1^ |
| Neotropical | *Atelopus chiriquiensis* | Costa Rica, Panama | 0.0 | Recreational activities^1^ |
| Neotropical | *Craugastor glaucus* | Mexico | 0.0 | Recreational activities^1^ |
| Neotropical | *Eleutherodactylus grandis* | Mexico | 0.0 | Recreational activities^1^ |
| Neotropical | *Eleutherodactylus poolei* | Cuba | 0.0 | Recreational activities^1^ |
| Neotropical | *Scinax alcatraz* | Brazil | 0.0 | Recreational activities^2^ |
| Neotropical | *Alsodes montanus* | Chile | 0.0 | Infrastructure and recreation^3^ |
| Neotropical | *Eleutherodactylus rivularis* | Cuba | 0.3 | Infrastructure^3^ |
| Neotropical | *Eleutherodactylus bartonsmithi* | Cuba | 0.4 | Recreational activities^2^ |
| Neotropical | *Eleutherodactylus schmidti* | Dominican Republic, Haiti | 0.4 | Recreational activities^2^ |
| Neotropical | *Atelopus zeteki* | Panama | 0.9 | Infrastructure^2^ |
| Neotropical | *Eleutherodactylus symingtoni* | Cuba | 1.4 | Infrastructure and recreation^3^ |
| Neotropical | *Eleutherodactylus tonyi* | Cuba | 1.5 | Infrastructure and recreation^3^ |
| Neotropical | *Eleutherodactylus blairhedgesi* | Cuba | 1.7 | Infrastructure and recreation^2^ |
| Neotropical | *Craugastor pozo* | Mexico | 1.8 | Infrastructure^1^ |
| Neotropical | *Eleutherodactylus bresslerae* | Cuba | 2.9 | Recreational activities^2^ |
| Neotropical | *Eleutherodactylus albipes* | Cuba | 3.2 | Recreational activities^2^ |
| Neotropical | *Eleutherodactylus turquinensis* | Cuba | 4.0 | Recreational activities^2^ |
| Neotropical | *Eleutherodactylus cubanus* | Cuba | 4.4 | Recreational activities^3^ |
| Neotropical | *Eleutherodactylus orientalis* | Cuba | 5.0 | Recreational activities^3^ |
| Neotropical | *Telmatobius culeus* | Bolivia, Peru | 5.1 | Recreational activities^1^ |
| Neotropical | *Craugastor megalotympanum* | Mexico | 5.9 | Recreational activities^1^ |
| Neotropical | *Holoaden bradei* | Brazil | 6.9 | Recreational activities^3^ |
| Neotropical | *Atelopus laetissimus* | Colombia | 7.6 | Infrastructure^1^ |
| Neotropical | *Mannophryne caquetio* | Venezuela | 10.4 | Recreational activities^1^ |
| Neotropical | *Prostherapis dunni* | Venezuela | 11.9 | Infrastructure^1^ |
| Neotropical | *Atelopus mucubajiensis* | Venezuela | 12.4 | Infrastructure^2^ |
| Neotropical | *Eleutherodactylus fuscus* | Jamaica | ? | Infrastructure^3^ |
| Neotropical | *Eleutherodactylus locustus* | Puerto Rico | ? | Infrastructure and recreation^3^ |
| Neotropical | *Eleutherodactylus alticola* | Jamaica | ? | Recreational activities^3^ |
| Neotropical | *Eleutherodactylus cavernicola* | Jamaica | ? | Recreational activities^3^ |
| Neotropical | *Eleutherodactylus juanariveroi* | Puerto Rico | ? | Recreational activities^2^ |
| Palearctic | *Rana holtzi* | Turkey | 0.0 | Infrastructure and recreation^3^ |
| Palearctic | *Odorrana wuchuanensis* | China | 0.0 | Recreational activities^2^ |
| Palearctic | *Glandirana minima* | China | ? | Infrastructure^1^ |

^a^ ? = proportion protected by tourism undetermined due to lack of financial data

^b 1^ = low impact, ^2^ = moderate impact, ^3^ = high impact
